# *Bacteroides* spp. promotes branched-chain amino acid catabolism in brown fat and inhibits obesity

**DOI:** 10.1016/j.isci.2021.103342

**Published:** 2021-10-24

**Authors:** Naofumi Yoshida, Tomoya Yamashita, Tatsunori Osone, Tetsuya Hosooka, Masakazu Shinohara, Seiichi Kitahama, Kengo Sasaki, Daisuke Sasaki, Takeshi Yoneshiro, Tomohiro Suzuki, Takuo Emoto, Yoshihiro Saito, Genki Ozawa, Yushi Hirota, Yasuyuki Kitaura, Yoshiharu Shimomura, Yuko Okamatsu-Ogura, Masayuki Saito, Akihiko Kondo, Shingo Kajimura, Takeshi Inagaki, Wataru Ogawa, Takuji Yamada, Ken-ichi Hirata

**Affiliations:** 1 Division of Cardiovascular Medicine, Department of Internal Medicine, Kobe University Graduate School of Medicine, 7-5-1 Kusunoki-cho, Chuo-ku, Kobe 6500017, Japan; 2 AMED-PRIME, Japan Agency for Medical Research and Development, 1-8-1 Inohana, Chuo-ku, Tokyo 1008152, Japan; 3 School and Graduate School of Bioscience and Biotechnology, Tokyo Institute of Technology, Tokyo 1528550, Japan; 4 Laboratory of Nutritional Physiology, School of Food and Nutritional Sciences/Graduate Division of Nutritional and Environmental Sciences, University of Shizuoka, Shizuoka 4228526, Japan; 5 Division of Epidemiology, Kobe University Graduate School of Medicine, Kobe 6500017, Japan; 6 The Integrated Center for Mass Spectrometry, Graduate School of Medicine, Kobe University, Kobe 6500017, Japan; 7 Department of Metabolic and Bariatric Surgery, Center for Obesity, Diabetes and Endocrinology, Chibune General Hospital, Osaka 5550034, Japan; 8 Graduate School of Science, Technology and Innovation, Kobe University, Kobe 6578501, Japan; 9 Bio Palette Co., Ltd., Kobe 6500047, Japan; 10 Division of Metabolic Medicine, Research Center for Advanced Science and Technology, The University of Tokyo, Tokyo 1538904, Japan; 11 Laboratory of Epigenetics and Metabolism, Institute for Molecular and Cellular Regulation, Gunma University, Gunma 3718512, Japan; 12 TechnoSuruga Laboratory Co., Ltd., Shizuoka 4240065, Japan; 13 Division of Diabetes and Endocrinology, Department of Internal Medicine, Kobe University Graduate School of Medicine, Kobe 6500017, Japan; 14 Laboratory of Nutritional Biochemistry, Department of Applied Biosciences, Graduate School of Bioagricultural Sciences, Nagoya University, Nagoya 4648601, Japan; 15 Department of Food and Nutritional Sciences, College of Bioscience and Biotechnology, Chubu University, Kasugai, Aichi 4878501, Japan; 16 Faculty of Veterinary Medicine, Hokkaido University, Sapporo 0600818, Japan; 17 Division of Endocrinology, Diabetes & Metabolism, Beth Israel Deaconess Medical Center, Harvard Medical School, Boston, MA 02215, USA

**Keywords:** Computer systems organization, Energy engineering, Energy systems

## Abstract

The gut microbiome has emerged as a key regulator of obesity; however, its role in brown adipose tissue (BAT) metabolism and association with obesity remain to be elucidated. We found that the levels of circulating branched-chain amino acids (BCAA) and their cognate α-ketoacids (BCKA) were significantly correlated with the body weight in humans and mice and that BCAA catabolic defects in BAT were associated with obesity in diet-induced obesity (DIO) mice. Pharmacological systemic enhancement of BCAA catabolic activity reduced plasma BCAA and BCKA levels and protected against obesity; these effects were reduced in BATectomized mice. DIO mice gavaged with *Bacteroides dorei* and *Bacteroides vulgatus* exhibited improved BAT BCAA catabolism and attenuated body weight gain, which were not observed in BATectomized DIO mice. Our data have highlighted a possible link between the gut microbiota and BAT BCAA catabolism and suggest that *Bacteroides* probiotics could be used for treating obesity.

## Highlights

• Gut microbiota regulated BAT BCAA catabolism
• *Bacteroides* promoted BAT BCAA catabolism and inhibited obesity
• *Bacteroides* suppressed BAT inflammation that contributed to BAT BCAA catabolic defect

## Graphical abstract

**Figure:**
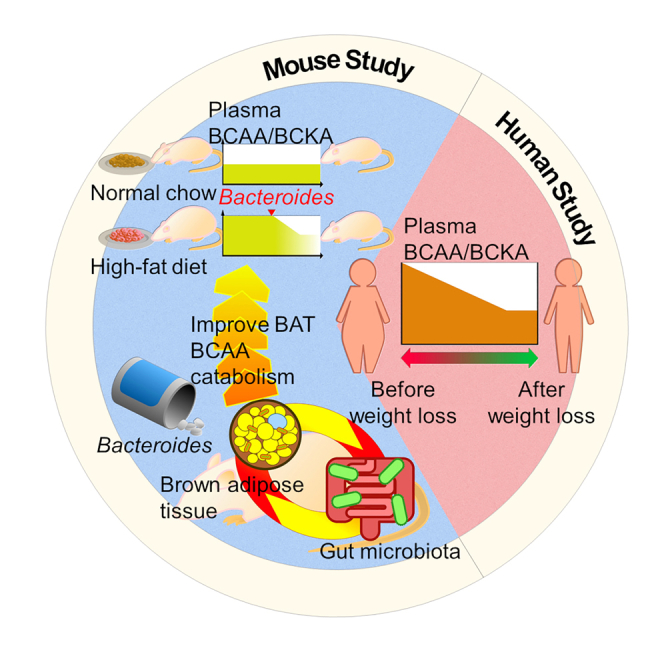

## Introduction

Obesity and its associated metabolic and cardiovascular disorders have seen a drastic increase in prevalence, with more than 600 million cases worldwide (Heymsfield and Wadden, 2017). Increasing evidence suggests a strong causal relationship between gut microbiota and obesity (Heymsfield and Wadden, 2017; Liu et al., 2017; Miyamoto et al., 2019; Turnbaugh et al., 2006, 2009); hence, gut microbial modulation could be an effective therapeutic strategy. However, our recent systematic review and meta-analysis, which included four randomized controlled trials, showed that uniform intervention with oral antibiotics does not alter the metabolic status of obese individuals, although it does affect their gut microbiota (Yoshida et al., 2020a). Considering this, exploring the role of specific gut bacteria involved in obesity to be able to exploit them as therapeutics is warranted. Generally, individuals with obesity are prone to dysbiosis, which is characterized by decreased gut bacterial diversity and richness (Liu et al., 2017; Turnbaugh et al., 2009) and exhibit a high *Firmicutes/Bacteroidetes* (F/B) ratio (Turnbaugh et al., 2006). *Bacteroides dorei* and *Bacteroides vulgatus*, belonging to the phylum Bacteroidetes, are dominant gut microbial species that are depleted in individuals with obesity (Liu et al., 2017), have an important role in maintaining a healthy gut ecosystem, play a protective role against atherosclerosis, and decrease fecal lipopolysaccharide activity, as we have previously reported (Emoto et al., 2016; Yoshida et al., 2018, 2020b). These findings support the encouraging prospect that obesity can be prevented by modulating the gut microbiome via supplementation of *B. dorei* and *B. vulgatus*.

Meanwhile, individuals with obesity were reported to have higher levels of circulating branched chain amino acids (BCAA) and branched-chain α-keto acids (BCKA) compared with lean individuals (Mardinoglu et al., 2018; Newgard et al., 2009), suggesting a potential causative role of BCAA and BCKA in the development of obesity. We and our collaborators have previously identified that brown adipose tissue (BAT) actively utilizes BCAA as an energy source and that cold stimuli markedly increase mitochondrial BCAA catabolism in BAT, leading to enhanced BCAA clearance in the circulation (Yoneshiro et al., 2019). However, whether BCAA catabolism in BAT is impaired in obesity and whether enhancing this catabolism can inhibit obesity remain unclear. Furthermore, environmental factors that regulate BCAA catabolism in BAT remain insufficiently understood. Although the expression of gut microbial genes related to BCAA catabolism has been reported to be associated with host insulin resistance (Pedersen et al., 2016), whether the gut microbiota, which is an important environmental factor involved in obesity, directly affect BCAA catabolism in BAT remains to be elucidated.

To address these knowledge gaps, we conducted a translational study using plasma and fecal samples from patients with obesity and a diet-induced obesity (DIO) mouse model. Here, we demonstrated that circulating BCAA and BCKA levels were significantly correlated with body weight and that BCAA catabolic defects in BAT have a causative role in obesity. *B. dorei* and *B. vulgatus* promoted BCAA catabolism in BAT and protected against obesity. Furthermore, *Bacteroides* probiotics increased the abundance of *Bacteroides* in the composite obese human fecal bacterial population in our culturing system. These results highlight gut microbial modulation by *B. dorei* and *B. vulgatus* as a potential strategy for treating obesity.

## Results

### Plasma BCAA and BCKA levels are correlated with body weight in humans and mice

**Figure 1:**
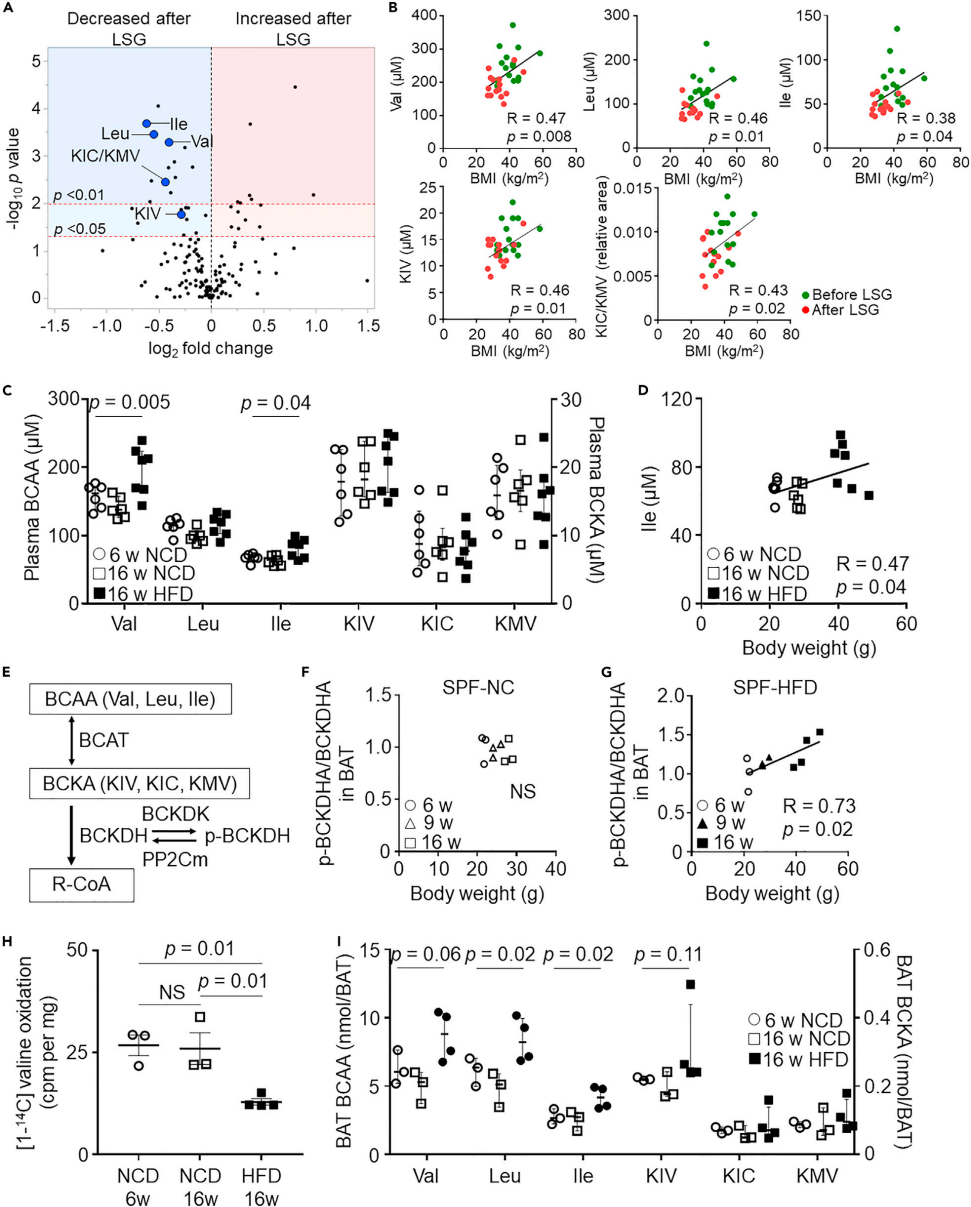
Association between BCAA catabolic defects in BAT and obesity (A, B) Data from 15 patients with obesity who underwent laparoscopic sleeve gastrectomy (LSG). (A) Plasma metabolite profiles before and after LSG. Volcano plot of the statistical significance against fold change. *n* = 15 per group. (B) Correlation between BMI and plasma BCAA (Val, Leu, Ile) and BCKA (KIV, KIC/KMV) levels. (C) Plasma BCAA and BCKA levels in mice fed NCD or HFD in indicated aged mice. (D) Correlation between body weight and plasma Ile levels in mice. (E) Schematic of enzymes and regulators involved in BCAA catabolism. (F, G) p-BCKDHA:BCKDHA ratio in specific-pathogen-free (SPF) mice fed NCD (F) or HFD (G) over time. (H) Valine oxidation in the BAT in the indicated groups. (I) BCAA and BCKA levels in BAT over time in SPF mice fed NCD or HFD. Data are shown as median ± interquartile range (25th to 75th percentile) (C, I); two-tailed paired Student's t test (A); Kruskal-Wallis test (C, I); Pearson's correlation coefficient (B, D, F, G). BCAT, branched-chain aminotransferase; BCKDH, branched-chain α-keto acid dehydrogenase; BCKDK, branched-chain α-keto acid dehydrogenase kinase; Ile, isoleucine; KIC, ketoisocaproic acid; KIV, ketoisovaleric acid; KMV, keto-beta-methylvaleric acid; Leu, leucine; LSG, laparoscopic sleeve gastrectomy; NS, not significant; p-BCKDH, phosphorylation of the BCKDH; PP2Cm, protein phosphatase 2C family member; Val, valine.

To determine whether plasma BCAA and BCKA can be used as a high-sensitivity metabolic hallmark that reflects body weight in humans, we first collected plasma samples from 15 Japanese patients with morbid obesity before and 3 months after laparoscopic sleeve gastrectomy (LSG). The mean age was 52.2 ± 6.5 years, and 80% were female. Three months after LSG, the body mass index (BMI) significantly decreased (before LSG 40.8 ± 6.6 kg/m^2^ versus after LSG 33.5 ± 6.1 kg/m^2^, p < 0 .001) along with decreased glycohemoglobin levels (Table S1). Plasma metabolomics using capillary electrophoresis time-of-flight mass spectrometry (CE-TOFMS), which can evaluate 916 ionic metabolites, identified 185 metabolites and several metabolite alterations, including significantly decreased BCAA (valine, leucine, isoleucine) and BCKA (alpha-ketoisovaleric acid [KIV], alpha-ketoisocaproic acid [KIC], and alpha-keto-beta-methylvaleric acid [KMV]) levels after LSG (Figure 1A; Table S2). A significantly positive correlation was observed between plasma BCAA and BMI and between plasma BCKA and BMI (Figure 1B). To further assess this, we used data from 11 nonobese patients (BMI 24.4 ± 3.1 kg/m^2^; Figure S1A) and compared plasma BCAA and BCKA levels with patients with obesity. We found that plasma valine and KIV levels in patients with obesity were higher than those of nonobese patients (Figure S1B). The results also confirmed the positive correlation between plasma valine and KIV levels and BMI (Figure S1C).

We next examined the plasma BCAA and BCKA levels in mice fed a normal-chow diet (NCD) or high-fat diet (HFD; 60% kcal fat). In NCD-fed mice, plasma BCAA and BCKA levels did not differ between 6 and 16 weeks of age (Figure 1C). In contrast, plasma valine and isoleucine levels in HFD-fed, 16-week-old mice were significantly increased compared with that of NCD-fed mice (Figure 1C). Furthermore, we observed a significantly positive correlation between plasma isoleucine levels and body weight, which was similar to the human data (Figure 1D). These results indicate that circulating BCAA and BCKA levels are closely linked to obesity and that BCAA and BCKA underlie the pathophysiology of obesity in both humans and mice.

### BCAA catabolic defects in BAT are associated with obesity

In BCAA catabolism, the irreversible decarboxylation of BCKA by the BCKA dehydrogenase (BCKDH) complex is a rate-limiting step (Figure 1E). Inactivation of the BCKDH E1α (BCKDHA) subunit by the BCKDH kinase (BCKDK) via the phosphorylation of BCKDHA (p-BCKDHA) suppresses BCAA catabolism (Figure 1E). As we reported, BCAA and BCKA are important energy sources for BAT thermogenesis, and BAT-specific defects in BCAA catabolism attenuate systemic BCAA clearance (Yoneshiro et al., 2019). Then we evaluated BAT BCAA catabolic activity under NCD and HFD conditions to elucidate whether obesity exacerbated BCAA catabolism in BAT.

In specific pathogen-free (SPF) mice fed an NCD, BCAA catabolic activity in BAT, as assessed by the p-BCKDHA to BCKDHA ratio, was not altered in 6-, 9-, and 16-week-old mice (Figure 1F). In contrast, HFD-fed SPF mice exhibited BCAA catabolic defects in BAT in a body-weight-dependent manner (Figure 1G). Valine oxidation in the BAT of 16-week-old mice fed an HFD significantly decreased compared with that in 6- and 16-week-old mice fed NCD (Figure 1H). These findings were consistent with the absolute quantification of BCAA and BCKA in BAT; the levels did not differ in NCD-fed, 6- and 16-week-old mice but were significantly higher in HFD-fed, 16-week-old mice (Figure 1I). Altogether, the results indicate that the HFD-induced BCAA catabolic defects in BAT are associated with obesity.

### BAT BCAA catabolism directly contributes to obesity

**Figure 2:**
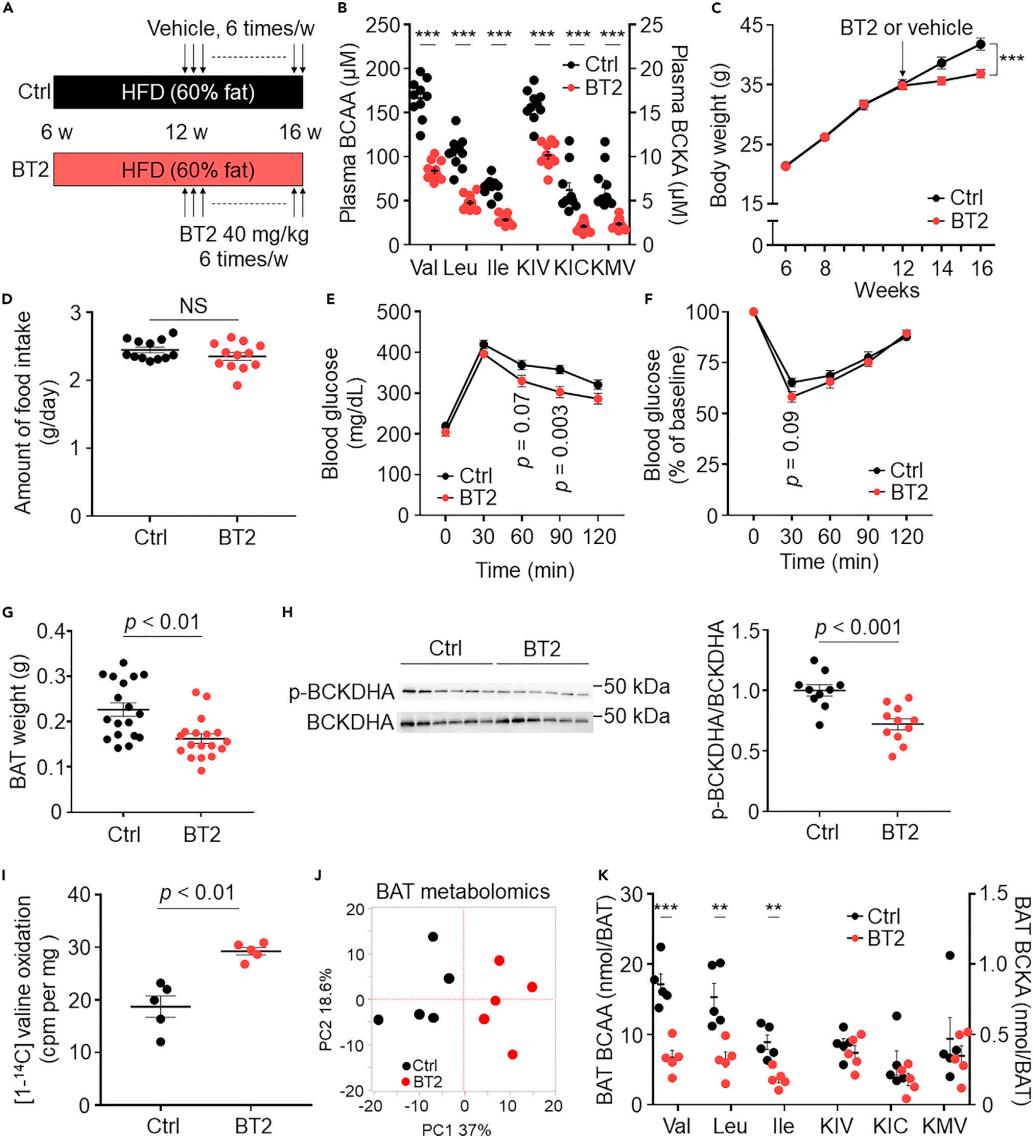
Pharmacological enhancement of BCAA catabolism inhibits obesity in DIO mice (A) Experimental design. (B) Plasma BCAA and BCKA levels in the indicated groups (Ctrl, vehicle; BT2, BT2 treatment). (C) Body weight changes. *n* = 18 per group. (D) Amount of food intake. (E) 1-g oral glucose-tolerance test. *n* = 10 (Ctrl) and 11 (BT2). (F) Insulin tolerance test. Insulin (1 U per kg body weight) was injected intraperitoneally. *n* = 12 (Ctrl) and 13 (BT2). (G) Weight of the BAT. (H) Representative results of immunoblotting and the ratio of p-BCKDHA:BCKDHA in the BAT. (I) Valine oxidation of the BAT in the indicated groups. (J) Principal component analysis (PCA) score plots of BAT metabolites in the indicated groups. (K) BAT BCKA and BCAA levels determined by LC-MS. Data are shown as mean ± SEM (B–I, K); two-tailed unpaired Student's t test (B, D, G, H, I, K); two-way repeated measures ANOVA (C) followed by post-hoc unpaired t test (E, F). ∗∗p < 0.01, ∗∗∗p < 0.001. BCKDHA; branched-chain ketoacid dehydrogenase subunit E1α; BT2, 3,6-dichlorobenzo[b]thiophene-2-carboxylic acid; DIO, diet-induced obesity; KIC, ketoisocaproic acid; KIV, ketoisovaleric acid; KMV, keto-beta-methylvaleric acid; NS, not significant; p-BCKDHA, phospho-BCKDHA.

Next, we modulated BCAA catabolism to investigate whether BCAA catabolic defects in BAT have a causative role in the progression of obesity and whether alleviation of these defects can inhibit obesity. We administered BT2 (3,6-dichlorobenzo[b]thiophene-2-carboxylic acid), a pharmacological BCAA catabolism enhancer, by oral gavage to DIO mice (Figure 2A). Four weeks after BT2 treatment, plasma BCAA and BCKA levels in the BT2 group were significantly reduced compared with those in the control mice (Figure 2B). Notably, BT2 treatment significantly inhibited the weight gain compared with control (Figure 2C), although the amount of HFD intake did not differ between the two groups (Figure 2D). Furthermore, BT2-treated mice showed significantly decreased systemic glucose intolerance and tended to decrease insulin resistance compared with control (Figures 2E and 2F). Although the weights of BAT, inguinal white adipose tissue (iWAT), and epididymal WAT (eWAT) in BT2-treated mice were significantly reduced compared with those in control (Figures 2G and S2A), BT2 treatment significantly reduced BCKDHA phosphorylation in only BAT (Figures 2H and S2B–S2D). Further, BT2-treated mice exhibited significantly increased valine oxidation in BAT than the control mice (Figure 2I). Nontargeted BAT metabolomics identified 306 metabolites and revealed that metabolites from BT2- or vehicle-treated mice were categorized into different clusters (Figure 2J, Table S3). By focusing on BCAA and BCKA, we observed that BT2-treated mice showed significantly reduced BAT BCAA levels (Figure 2K).

**Figure 3:**
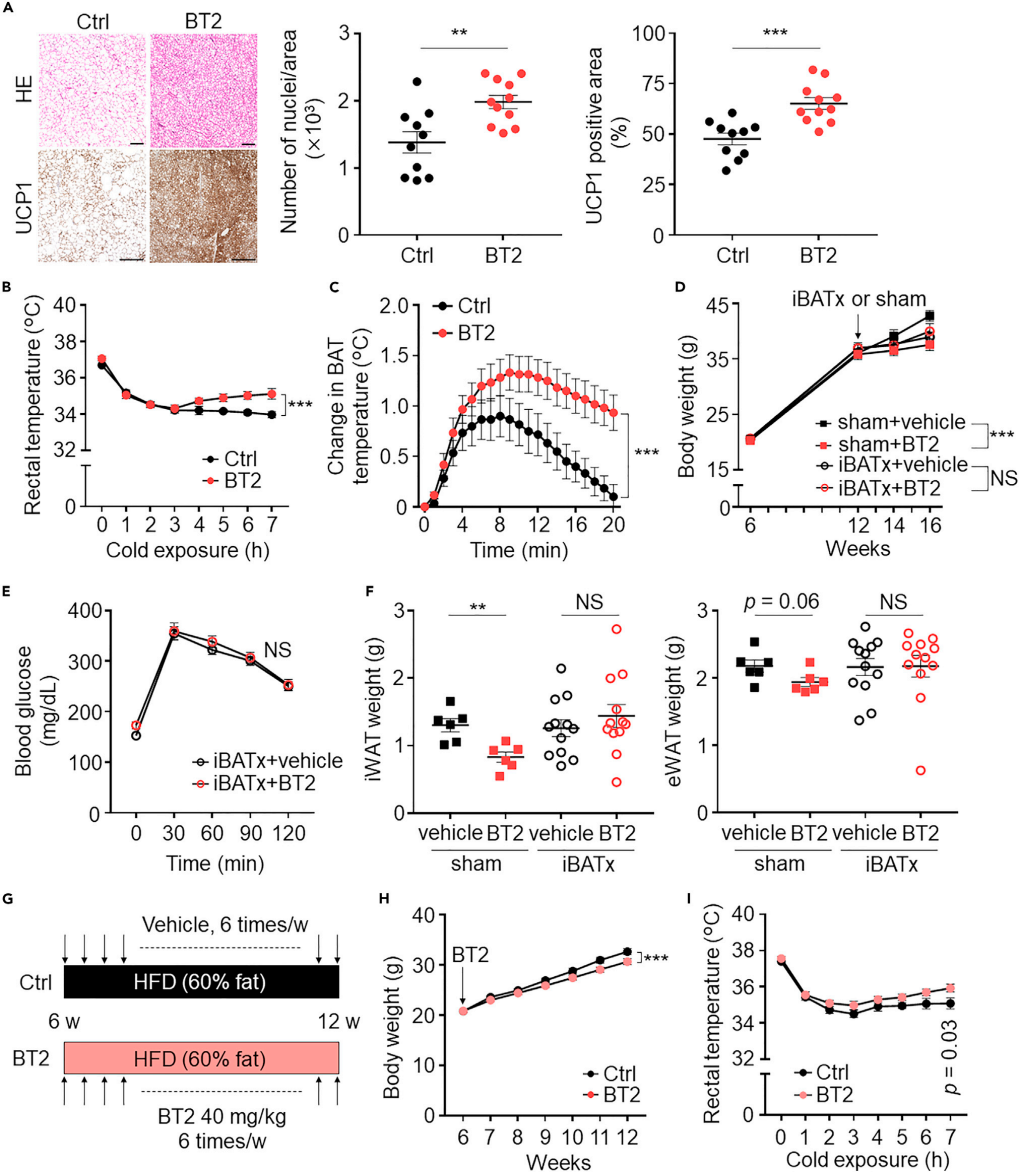
BAT BCAA catabolic defects directly contribute to obesity in DIO mice (A) Representative HE and UCP1 staining of BAT. Scale bar, 200 μm. (B) Rectal core body temperature following cold exposure at 8°C. *n* = 12 per group. (C) Change in BAT temperature following treatment with noradrenaline. *n* = 6 per group. (D) Body weight changes in the indicated groups. Resection of interscapular BAT or sham operation was performed on 12-week-old HFD-fed mice (HFD from 6 weeks) that were randomly divided into four groups. i.e., sham + vehicle (*n* = 6), sham + BT2 (*n* = 6), iBATx + vehicle (*n* = 12), and iBATx + BT2 (*n* = 12). (E) 1-g oral glucose-tolerance test. *n* = 6 per group. (F) Weights of the iWAT and eWAT in the indicated groups. (G) Experimental design. (H) Body weight changes in the indicated groups. *n* = 12 per group. (I) Rectal core body temperature following cold exposure at 8°C at 12 weeks of age. *n* = 8 per group. Data are shown as mean ± SEM (A–F, H, I); two-tailed unpaired Student's t test (A, F); two-way repeated measures ANOVA (E, H) followed by post-hoc unpaired t test (I). ∗∗p < 0.01, ∗∗∗p < 0.001. BAT, brown adipose tissue; BT2, 3,6-dichlorobenzo[b]thiophene-2-carboxylic acid; iBATx, interscapular BATectomy; KIC, ketoisocaproic acid; KIV, ketoisovaleric acid; KMV, keto-beta-methylvaleric acid; NS, not significant; UCP1, uncoupling protein 1; WAT, white adipose tissue.

Moreover, histological analysis of BAT revealed a significantly increased nuclei number and UCP1 expression (Figure 3A). Notably, the core body temperature of BT2-treated mice was significantly higher than that of the control mice following cold exposure or noradrenaline injection (Figures 3B and 3C), suggesting that enhanced BAT BCAA catabolism accelerates BAT thermogenesis. To examine whether the reduction in body weight gain following BT2 treatment is BAT dependent, we surgically removed the interscapular BAT (iBATx) in DIO mice. Subsequently, the decrease in body weight gain following BT2 treatment was diminished in iBATx DIO mice, whereas sham DIO mice treated with BT2 exhibited significant inhibition of body weight gain compared with control (Figure 3D). We also observed that iBATx DIO mice treated with BT2 did not exhibit amelioration of systemic glucose intolerance (Figure 3E). Furthermore, in iBATx DIO mice, BT2 treatment did not alter the weight of iWAT and eWAT (Figure 3F). These results indicate that the improvement in metabolic health after BT2 treatment is BAT dependent. We further investigated whether BT2 can prevent obesity in DIO mice by gavaging mice with BT2 at 6 weeks of age (Figure 3G). BT2-treated DIO mice exhibited a significant decrease in body weight gain (Figure 3H) and higher core body temperatures after cold exposure at 8°C compared with control mice (Figure 3I). Altogether, the results indicate that BCAA catabolic defects in BAT directly contribute to obesity and that enhancement of BCAA catabolic activity improves BAT thermogenesis as well as decreases in systemic glucose intolerance and adiposity and protects against obesity in DIO mice.

### *Bacteroides* treatment improves BAT BCAA catabolism and inhibits obesity in DIO mice

**Figure 4:**
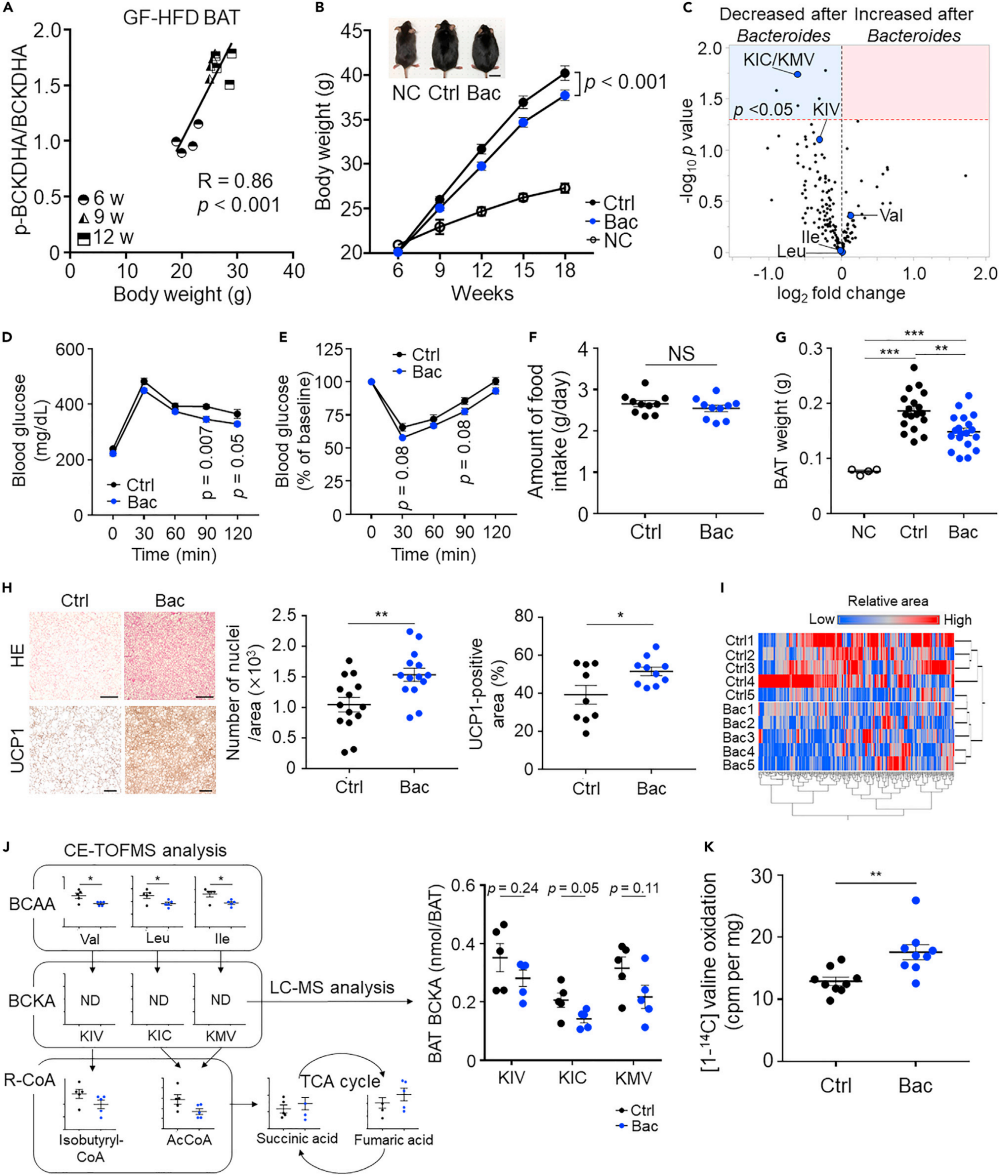
Impact of *Bacteroides* treatment on obesity and BAT BCAA catabolism in DIO mice (A) The p-BCKDHA:BCKDHA ratio over time in HFD-fed germ-free (GF) mice. (B) Body weight changes in the indicated groups and representative photos of the indicated mice. Scale bar, 2 cm. *n* = 4 (NC, normal chow), 18 (Ctrl, high-fat diet [HFD] + vehicle), and 19 (Bac, HFD + *Bacteroides* treatment). (C) Comparison of the plasma metabolite profiles of mice treated with *Bacteroides* or vehicle. Volcano plot of the statistical significance against fold change. *n* = 5 per group. (D) 1-g oral glucose-tolerance test. *n* = 14 per group. (E) Insulin tolerance test. *n* = 8 per group. (F) Amount of food intake in the indicated groups. (G) Brown adipose tissue (BAT) weight. (H) Representative hematoxylin and eosin (HE) staining (scale bar, 200 μm) and uncoupling protein 1 (UCP1) staining (scale bar, 100 μm.) of BAT. Number of nuclei and UCP1-positive area are compared between the indicated groups. (I) Heat maps of the cluster analysis performed using nontargeted BAT metabolomics data. Horizontal axis, 264 metabolites; vertical axis, sample name. *n* = 5 per group. (J) Relative area of metabolites associated with the branched chain amino acid (BCAA) catabolic pathway in (I). BCKA levels were absolutely quantified using LC-MS. (K) Valine oxidation of the BAT. Data are shown as mean ± SEM; ∗p < 0.05, ∗∗p < 0.01, ∗∗∗p < 0.001, NS, not significant. Pearson's correlation coefficient (A); two-way repeated measures ANOVA (B) followed by post-hoc unpaired t test (D, E); two-tailed unpaired Student's t test (C, F, H, J, K); one-way ANOVA followed by Tukey's post-hoc test (G). GF, germ-free; KIC, ketoisocaproic acid; KIV, ketoisovaleric acid; KMV, keto-beta-methylvaleric acid; N.D., not detected.

Of note, HFD-fed germ-free mice exhibited a drastic increase in the p-BCKDHA to BCKDHA ratio in the BAT in a body-weight-dependent manner (Figure 4A), suggesting a previous unknown association between the gut microbiota and BCAA catabolism in BAT and indicating that the gut microbiota is a key protective filter that controls BAT BCAA catabolism under HFD conditions. We thus explored the impact of *B. dorei* and *B. vulgatus* on obesity and its interaction with BAT BCAA catabolism to determine whether *Bacteroides* treatment strengthens the protective effects of gut microbiota on BCAA catabolic defects in BAT under obese condition. HFD-fed, 6-week-old male mice were gavaged with *B. dorei* and *B. vulgatus* for 12 weeks (Bac), after which their body weights and metabolic parameters were compared with those of the NCD-fed mice or HFD-fed mice that received a vehicle (culture medium; control) (Figure S3A). Surprisingly, gavage with *Bacteroides* significantly inhibited obesity and weight gain (Figure 4B). We also compared plasma metabolite concentrations between *Bacteroides*-gavaged mice and control using CE-TOFMS. A total of 195 metabolites were identified, among which the concentrations of KIC/KMV were significantly decreased and those of KIV were near-significantly decreased in *Bacteroides*-gavaged mice compared with those in control mice (Figure 4C; Table S4). Furthermore, compared with the control, *Bacteroides*-gavaged mice exhibited significantly decreased systemic glucose intolerance and near-significant improvement in insulin resistance, as revealed by the results of the 1-g oral glucose tolerance test and insulin tolerance test (Figures 4D and 4E). The amount of food intake plasma lipid levels and fecal short-chain fatty acid levels did not differ between *Bacteroides*-gavaged and control mice (Figures 4F, S3B, and S3C). *Bacteroides*-gavaged mice also exhibited significantly low inguinal fat mass with smaller-sized adipocyte cells (Figures S4A and S4B). Similarly, the epididymal fat tissue exhibited smaller adipocyte cells and reduced macrophage accumulation and fibrosis (Figures S4A and S4C). BAT weight was significantly lower in *Bacteroides*-treated mice than that in control mice (Figure 4G). Histological analysis of BAT revealed significantly increased nuclei number and uncoupling protein 1 (UCP1) expression in *Bacteroides*-treated mice compared with control mice (Figure 4H). Nontargeted BAT metabolomics using CE-TOFMS identified 264 metabolites and differences in metabolic characteristics between the two groups, as indicated by hierarchical cluster analyses (Figure 4I; Table S5). By focusing on the BCAA catabolic pathway, we found significantly decreased BCAA levels and lower BCKA and R-CoA (isobutyryl-CoA and acetyl-CoA) levels, whereas the levels of TCA intermediates tended to increase in *Bacteroides*-treated mice compared with those in the control (Figure 4J). Improved BAT BCAA catabolism in *Bacteroides*-treated mice was also confirmed using the BAT valine oxidation assay, which revealed significantly increased valine oxidation in mice treated with *Bacteroides* compared with that in control mice (Figure 4K).

16S rDNA amplicon sequencing revealed changes in the gut microbiota at the phylum and genus level (Figures S5A and S5B). Principal component analysis showed that the three groups differed in the abundance of gut bacteria (Figure S5C). A decreased F/B ratio and increased bacterial number and α-diversity in response to *Bacteroides* treatment compared with control were observed (Figures S5D–S5F). The abundance of *B. dorei* and *B. vulgatus* was also significantly increased in *Bacteroides*-gavaged mice (Figure S5G). As gut microbiota could potentially utilize BCAA (Pedersen et al., 2016), we evaluated the functions of gut microbe genes using PICRUSt. The PICRUSt analysis revealed that the expression levels of the BCAA degradation pathway did not differ between *Bacteroides*-treated mice and control mice (Figure S5H), indicating that gut microbiota did not contribute to decreased plasma BCKA levels after *Bacteroides* treatment.

**Figure 5:**
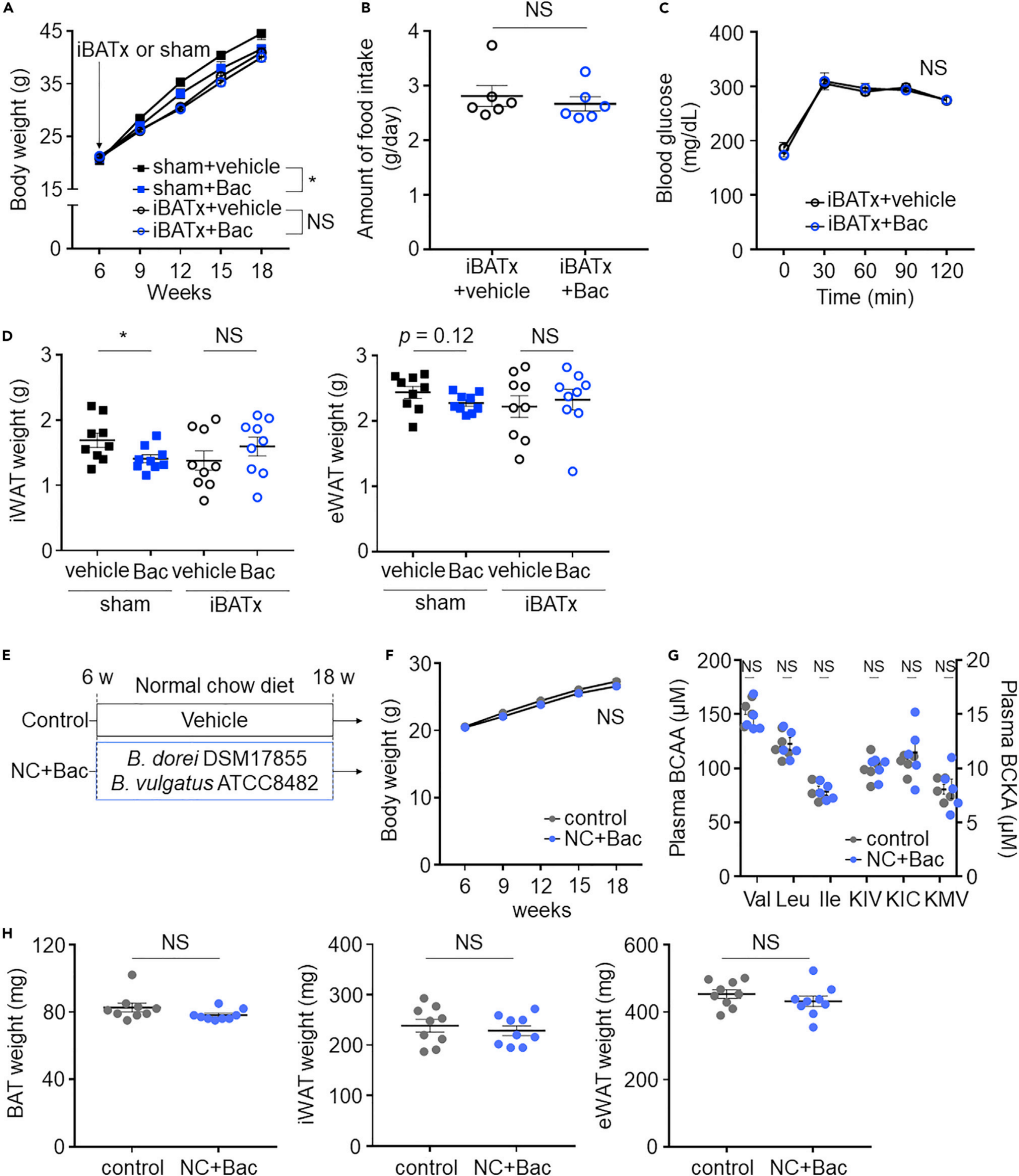
iBATx DIO mice treated with *Bacteroides* did not exhibit inhibition of body weight (A) Body weight changes in the indicated groups. Resection of interscapular BAT or sham operation was performed on 6-week-old HFD-fed mice that were randomly divided into four groups, i.e., sham + vehicle (*n* = 9), sham + Bac (*n* = 9), iBATx + vehicle (*n* = 17), and iBATx + Bac (*n* = 17). (B) 1-g oral glucose-tolerance test. *n* = 9 per group. (C) Amount of food intake. (D) Weights of iWAT and eWAT. (E) Experimental design. (F) Body weight changes in the indicated groups. *n* = 9 per group. (G) Plasma BCAA and BCKA levels. (H) Weights of the BAT, iWAT, and eWAT. Data are shown as mean ± SEM. Two-way repeated measures ANOVA (A, C, F) and two-tailed unpaired Student's t test (B, D, G, H). BAT, brown adipose tissue; Ile, isoleucine; KIC, ketoisocaproate; KIV, ketoisovalerate; KMV, ketoisoleucine; Leu, leucine; NS, not significant; Val, valine; WAT, white adipose tissue.

To determine whether the inhibition of obesity and improved glucose intolerance after *Bacteroides* treatment is BAT dependent, we gavaged HFD-fed iBATx mice with *Bacteroides*. The body weight gain in iBATx mice treated with *Bacteroides* or vehicle did not differ, whereas sham mice treated with *Bacteroides* exhibited significant inhibition of body weight gain (Figure 5A). The amount of food intake did not differ between the two groups (Figure 5B). We also observed that iBATx DIO mice treated with *Bacteroides* did not exhibit an amelioration of systemic glucose intolerance (Figure 5C). Importantly, *Bacteroides* treatment in iBATx DIO mice did not alter the weight of iWAT and eWAT (Figure 5D). These findings suggest that *Bacteroides* treatment enhances BCAA catabolism in BAT and protects against obesity, consequently reducing WAT weight.

These findings indicate that *Bacteroides* treatment alleviates HFD-induced BAT BCAA catabolic defects and improves metabolic health in DIO mice. Indeed, in NCD-fed mice, which did not manifest BAT BCAA catabolic defects (Figure 1F), *Bacteroides* treatment did not attenuate the body weight gain, decrease plasma BCAA and BCKA levels, and decrease BAT and WAT weight (Figures 5E–5H).

### *Bacteroides* treatment suppresses inflammation in BAT

**Figure 6:**
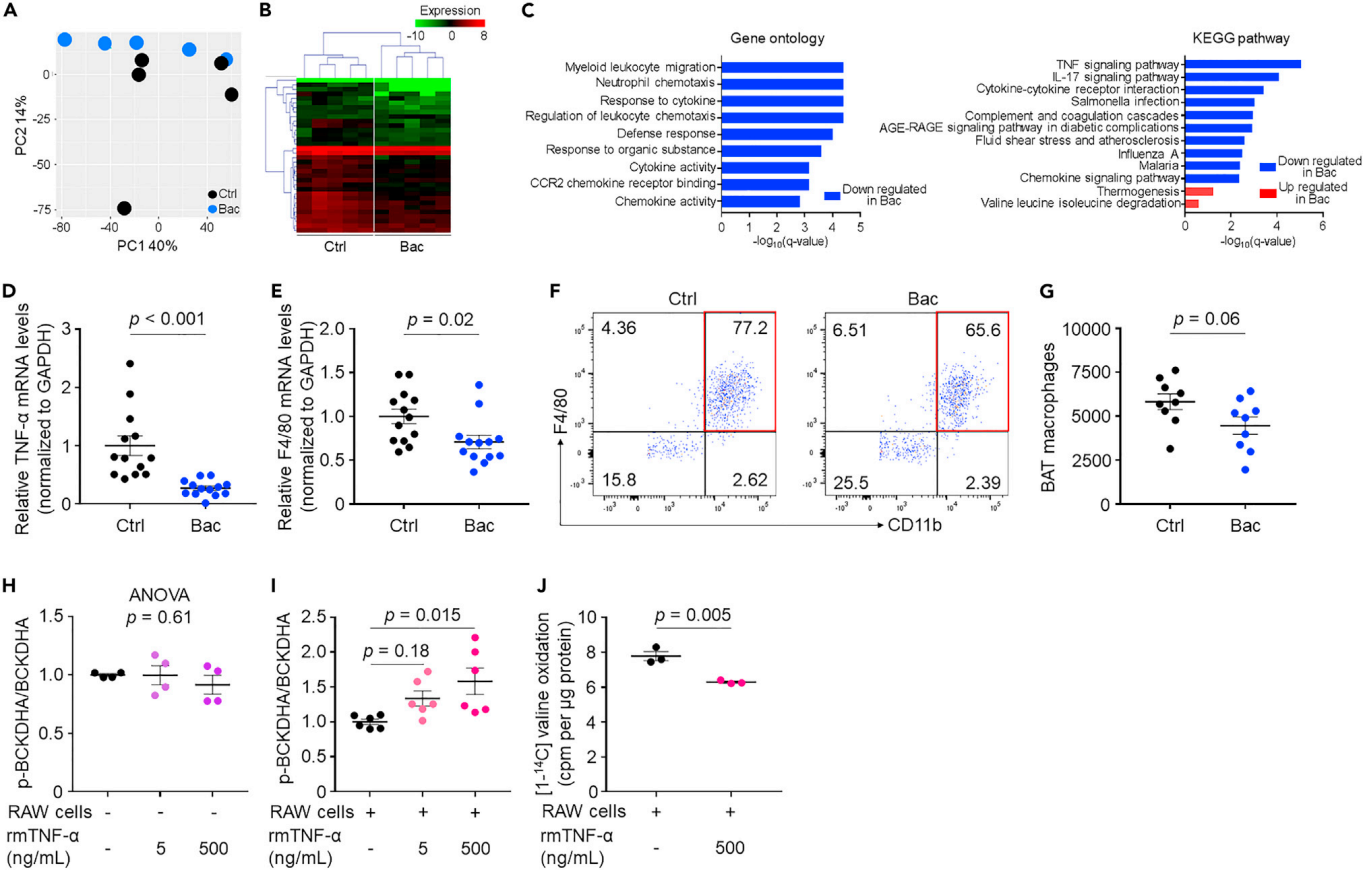
*Bacteroides* treatment suppresses inflammation, which plays a key role in BAT BCAA catabolism (A–C) BAT was collected at 18 weeks of age for RNA sequencing. *n* = 5 per group. (A) Principal component analysis (PCA) score plots. (B) Heat maps of cluster analysis showing the expression of differentially expressed genes in BAT of the indicated groups, with a dendrogram showing the clustering of genes and samples. (C) Top terms showing enrichment based on −log_10_ (q-value) from gene ontology enrichment and KEGG pathway analyses. (D and E) Real-time PCR of BAT TNF-α (D) and F4/80 (E) mRNA levels. (F) Analysis of macrophages in BAT by flow cytometry. Red square indicates CD11b^+^F4/80^+^ cells among the CD45^+^Ly6G^−^ population. (G) Number of macrophages (CD45^+^Ly6G^−^CD11b^+^F4/80^+^) in BAT. (H and I) The p-BCKDHA:BCKDHA ratio in HB2 cells stimulated with or without rmTNF-α (H) and co-cultured with RAW cells (I). (J) Valine oxidation in HB2 cells co-cultured with RAW cells and stimulated with or without rmTNF-α. Data are shown as the mean ± SEM; two-tailed unpaired Student's t test (D, E, G, J); one-way ANOVA (H) followed by Tukey's post-hoc test (I). BCKDHA; branched-chain ketoacid dehydrogenase subunit E1α; KEGG, Kyoto Encyclopedia of Genes and Genomes; p-BCKDHA, phospho-BCKDHA; rmTNF-α, recombinant murine tumor necrosis factor alpha.

To gain functional and mechanistic insights into the effects of *Bacteroides* on BAT BCAA catabolism, we performed RNA-seq; both groups exhibited distinct clusters (Figures 6A and 6B). Gene ontology enrichment and KEGG pathway analyses identified that most differentially expressed genes belonged to inflammatory processes (Figure 6C), which play pivotal roles in BAT functions (Alcalá et al., 2017; Nisoli et al., 2000). Although not statistically significant, “thermogenesis” and “valine, leucine, and isoleucine degradation” pathways were upregulated in *Bacteroides* mice (Figure 6C). We observed a marked change in the tumor necrosis factor (TNF) signaling pathway using KEGG pathway analysis (Figure 6C) and confirmed that the mRNA levels of TNF-α were significantly lower in *Bacteroides*-treated mice than in those in control mice (Figure 6D). As macrophages are known to mainly secrete TNF-α, we evaluated mRNA F4/80 levels in BAT and found significantly lower expression in *Bacteroides*-treated mice than that in control mice (Figure 6E). We further confirmed near-significant decrease in F4/80^+^ macrophage (CD45^+^Ly6G^−^CD11b^+^F4/80^+^) accumulation in the BAT of *Bacteroides*-treated mice compared with that in control mice using fluorescence-activated cell sorting analysis (Figures 3F, 3G, S6A, and S6B).

### Macrophages and TNF-α play a key role in BAT BCAA catabolism *in vitro*

We next examined the role of TNF-α and macrophages in BAT BCAA catabolism. HB2 brown adipocytes stimulated with recombinant TNF-α did not show an increased p-BCKDHA:BCKDHA ratio; however, the ratio increased in a co-culture system of HB2 brown adipocytes and RAW264 macrophages following TNF-α stimulation compared with no stimulation (Figures 6H and 6I). Furthermore, valine oxidation in HB2 brown adipocytes stimulated with RAW264 macrophage cells and TNF-α was significantly reduced compared with that observed in the absence of TNF-α stimulation (Figure 6J). Thus, mechanistically, inhibition of macrophage activation and TNF-α production may be involved in improved BAT BCAA catabolism following *Bacteroides* treatment.

### *Bacteroides* probiotics increased *Bacteroides* spp. abundance in the KUHIMM

**Figure 7:**
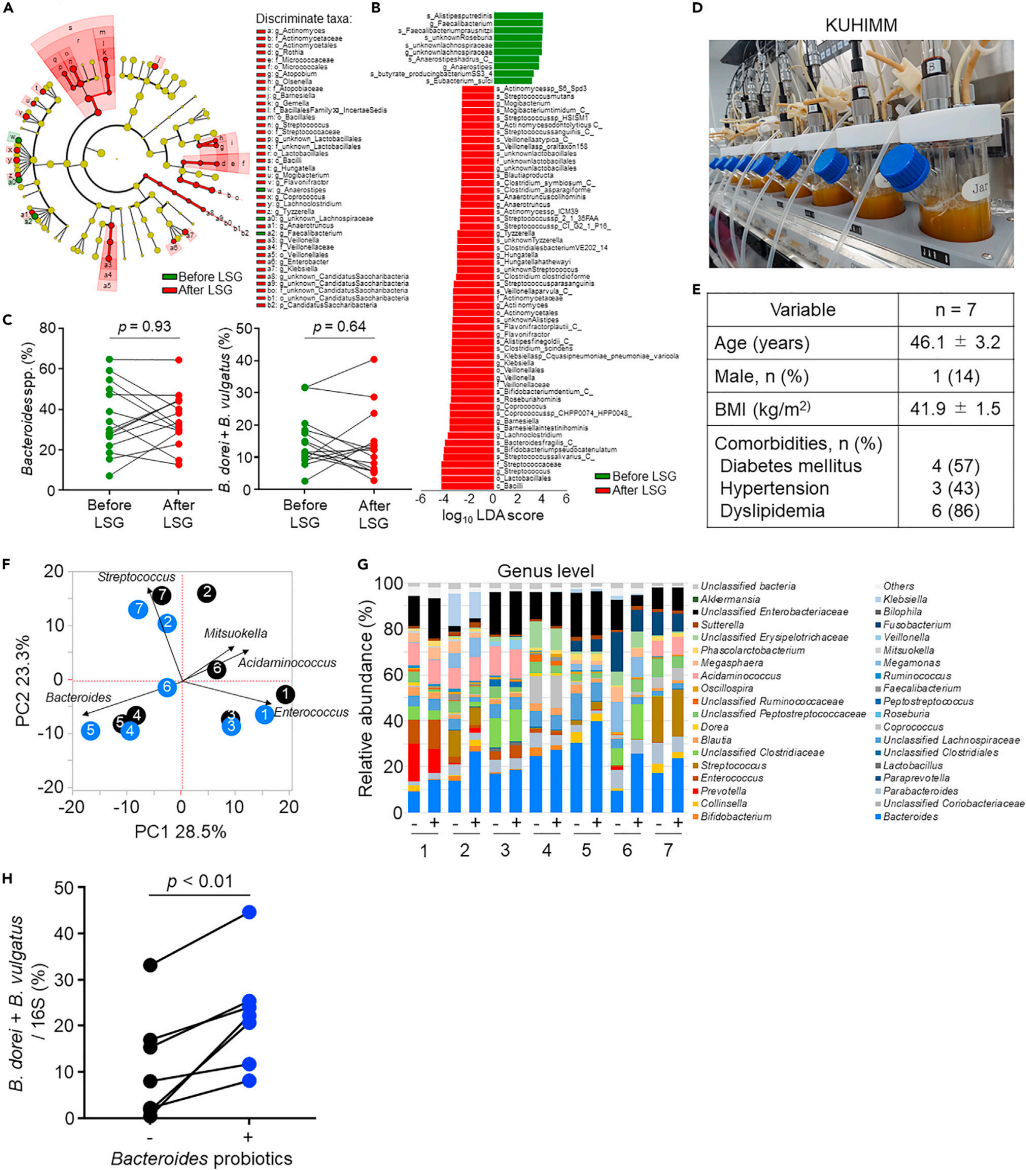
Effect of LSG or *Bacteroides* probiotics on the abundance of *Bacteroides* in the human gut microbiota (A–C) Metagenome-based gut microbial composition of 15 patients with obesity who underwent LSG. Sequence data were annotated using mOTU2. (A) Cladogram of the gut microbiota. Each dot represents a taxonomic hierarchy. The green and red dots indicate significant enrichment in the indicated groups (LEfSe: p < 0.05, q < 0.1, linear discriminant analysis [LDA] > 2). Yellow dots represent nonsignificant differences. (B) Relative abundance of the gut microbiota according to LDA score. (C) Relative abundance of *Bacteroides* spp., *B. dorei*, and *B. vulgatus*, before and after LSG. (D–H) Alteration of gut microbiota after *Bacteroides* supplementation in KUHIMM. (D) KUHIMM is a single-batch fermentation system composed of eight independent and parallel anaerobic culturing vessels. (E) Characteristics of patients whose feces were employed in the KUHIMM. Data are shown as mean ± SD or n (%). (F) Sequencing of the V3–V4 regions of bacterial 16S rRNA from fecal sample cultures collected from seven patients. PCA score plots at the genus level with (blue) or without (black) *Bacteroides* probiotics. Only genera with large weights in principal coordinate analysis are shown. (G) Relative abundance of the gut microbiota at the genus level of fecal sample cultures from seven patients with (+) or without (−) *Bacteroides* probiotics. (H) Relative abundance of *B. dorei* and *B. vulgatus* (percentage of total gut microbiota) with (blue) or without (white circle) *Bacteroides* probiotics. Two-tailed paired t test (C, H). LSG, laparoscopic sleeve gastrectomy; KUHIMM, Kobe University Human Intestinal Microbiota Model.

Finally, we compared the gut microbiota before and after LSG to evaluate alterations in the abundance of *B. dorei* and *B. vulgatus* (see Figures S7A and S7B for an overview of the metagenomic analysis pipeline). The cladogram generated with linear discriminant analysis (LDA) effect size analysis and LDA plot illustrated several significant alterations in the gut microbiota before and after LSG (Figures 7A, 7B, S7C and S7D). However, the abundance of *Bacteroides* spp., *B. dorei* and *B. vulgatus*, was not increased after LSG (Figures 7C and S7E), indicating that we can expect additional body weight reduction in patients with obesity if the abundance of *B. dorei* and B. *vulgatus* in the gut is increased. To determine whether probiotics can increase *Bacteroides* in the human gut, we used a single-batch anaerobic culturing system, Kobe University Human Intestinal Microbiota Model (KUHIMM; Figure 7D) (Takagi et al., 2016; Yoshida et al., 2019, 2020b), and cultured feces from seven obese individuals with *Bacteroides* probiotics (Figure 7E). KUHIMM can simulate the human gut microbiota metagenomically and metabolically, thereby facilitating evaluation of the impact of probiotics on gut microbiota before clinical trials; Figures 7F and 7G show various responses of the gut microbiota following *Bacteroides* probiotics supplementation in KUHIMM. Notably, *Bacteroides* probiotics significantly increased the abundance of *B. dorei* and *B. vulgatus*, even in the composite human bacterial population (Figure 7H). These findings support *Bacteroides* probiotics as a potential therapeutic strategy for treating obesity.

## Discussion

Higher plasma BCAA and BCKA levels in individuals with obesity, compared with lean controls, have been previously reported (Newgard et al., 2009). Furthermore, using RISC (Hills et al., 2004) and METSIM (Stancáková et al., 2009) data, plasma BCAA and BCKA levels were reported to be significantly correlated with BMI (Mardinoglu et al., 2018). Moreover, the expression of gut microbial genes related to BCAA biosynthesis is significantly associated with obesity-related metabolic health (Pedersen et al., 2016). Thus, elucidating the causality and mechanism underlying the increased BCAA and BCKA levels in obesity will better illustrate its pathophysiology and provide insights that enable the development of a breakthrough therapeutic strategy against obesity. Here, we demonstrated that impaired BCAA catabolism in BAT plays a role in obesity progression and that enhancement of this catabolism inhibits obesity. Further, we also elucidated the role of the gut microbiota in BAT BCAA catabolism and its association with obesity.

The primary findings of the present study are as follows: (1) BCAA catabolic defects in BAT occurred in a body-weight-dependent manner, and pharmacological enhancement of BAT BCAA catabolism inhibited obesity; (2) gavaging with *Bacteroides* spp. alleviated obesity-induced BCAA catabolic defects in BAT and inhibited obesity; (3) gavaging with *Bacteroides* spp. inhibited macrophage accumulation and TNF-α production in BAT, which could be involved in BAT BCAA catabolism; and (4) *Bacteroides* probiotics increased *Bacteroides* abundance in the composite human bacterial population in our fecal culturing system. Overall, our findings provide insights into the roles of gut microbiota in BCAA catabolism as well as obesity prevention and management. In addition, the results further support the notion that elevated plasma BCAA and BCKA levels are metabolic hallmarks of obesity in the clinic.

We have previously reported that BCAA catabolism in BAT is mediated by SLC25A44 for thermogenesis and that BAT plays an important role in systemic BCAA clearance and protects against obesity and insulin resistance (Yoneshiro et al., 2019). The BCAA catabolic pathway is genetically associated with obesity-associated insulin resistance in humans (Zhou et al., 2019); however, the association between the extent of impaired BCAA catabolism in BAT and obesity severity remains uncertain and the acquired factors that affect BCAA catabolism warrant further investigation. Herein, we showed that BAT BCAA catabolism is impaired in a body-weight-dependent manner. In addition, we showed that germ-free mice fed an HFD exhibited drastic BCAA catabolic defects in BAT as assessed by the p-BCKDHA/BCKDHA ratio. This is consistent with previous findings showing that plasma KIC/KMV levels are higher in germ-free mice than in SPF mice (Matsumoto et al., 2017). This impaired BAT BCAA catabolism in germ-free mice may be a plausible explanation for the impaired BAT thermogenesis in mice with microbial depletion (Li et al., 2019). We also demonstrated that gut microbial modulation by supplementation of *Bacteroides* spp. reduced BCAA and BCKA levels in BAT, which strongly suggests that *Bacteroides* spp. play a beneficial role in BCAA catabolism in BAT. Mechanistically, our data suggest that cell-to-cell contacts between brown adipocytes and macrophages stimulated with TNF-α are involved in BAT BCAA catabolic defects. Although we did not clarify the mechanism by which *Bacteroides* treatment suppresses BAT inflammation in DIO mice, our previous findings revealed a similar phenotype; TNF-α production and macrophages accumulation are inhibited after gavaging with *B. dorei* and *B. vulgatus* in apolipoprotein-E-deficient mice (Yoshida et al., 2018). We also showed that *Bacteroides* lipopolysaccharide induce relatively low-level proinflammatory cytokine production due to the unique lipopolysaccharide structure compared with that of *Escherichia coli* (Yoshida et al., 2020b). Taken these findings into consideration, anti-inflammatory effects following *Bacteroides* treatment may be a universal phenomenon. As BAT possesses the highest BCAA catabolic capacity in our organs (Neinast et al., 2019), elucidating this previously unknown link between gut microbiota and BAT BCAA catabolism as well as local interactions between BAT macrophages and BCAA catabolic defects can help provide novel therapeutic strategies for treating obesity via gut microbiota modulation. In addition, the role of macrophages in BAT is insufficiently understood. Our results could clarify the role of BAT macrophages and subsequently promote the functional analysis of BAT macrophages.

Several studies have shown that the abundance of *Bacteroides* spp*.*, such as *B. intestinalis*, *B. ovatus*, *B. thetaiotaomicron*, and *B. uniformis*, is low in obese individuals (Castaner et al., 2018; Ejtahed et al., 2020; Hu et al., 2015; Liu et al., 2017; López-Contreras et al., 2018); is negatively correlated with fasting blood glucose and glycohemoglobin levels in humans (Hermes et al., 2020; Liu et al., 2017); and inhibits obesity in DIO mice (Gauffin Cano et al., 2012; Liu et al., 2017; Yang et al., 2017). From a clinical perspective, as *B. dorei* and *B. vulgatus* are the dominant species within *Bacteroides* spp*.* and as they exhibit a protective role against atherosclerosis (Yoshida et al., 2018), we believe that our results provide new insights into the functions of *B. dorei* and *B. vulgatus.* Our results also show that the strategy of replenishment of these bacteria has great potential for treating obesity. We are in the process of isolating *B. dorei* and *B. vulgatus* from numerous individuals to select *Bacteroides* strains that have potential inhibitory effects with respect to obesity.

Recently, BAT has attracted significant attention as a modulator of metabolic and cardiovascular diseases such as diabetes, heart failure, and coronary artery disease (Becher et al., 2021; Connell et al., 2021). Although the gut microbiota is also involved in these diseases, gut microbiota-BAT interactions remain to be defined. Thus, we believe that our findings make a significant contribution to future research by addressing this knowledge gap. In summary, we identified the gut microbiota as an important environmental factor regulating BCAA catabolism in BAT and that *Bacteroides* spp. promote BCAA catabolism in BAT and protect against obesity.

### Limitations of the study

The following limitations of this study have to be considered. First, assessment for calorie expenditure or oxygen consumption rates may help to understand the impact of *Bacteroides* spp. on systemic energy balance and BAT mitochondrial metabolism. We did not perform these assays and thereby our results indicated relatively weak evidence in point of functional characterization after *Bacteroides* treatment in DIO mice. However, our BCAA oxidation assay clearly revealed that *Bacteroides* treatment promoted BAT BCAA catabolism, which have critical role in the pathophysiology of obesity. Second, we did not investigate whether long-term cold exposure alter the abundance of *B. dorei* and *B. vulgatus*. This result may support our idea that *B. dorei* and *B. vulgatus* have impact on BAT BCAA catabolism and BAT thermogenesis in cold stimulus. Third, we did not mention macrophage phenotype that regulate BAT BCAA catabolism. Single-cell analysis of BAT will help elucidate the impact of BAT macrophages on immunometabolism in BAT or identify more specific macrophage types that regulate BCAA catabolism. Further studies are warranted to proof our research concepts that the gut microbiota is one of the key environmental factors that regulate BAT BCAA catabolism.

## STAR★Methods

### Key resources table

**Table:** 

REAGENT or RESOURCE	SOURCE	IDENTIFIER
**Antibodies**		

Anti-LAMP-2 (M3/84) antibody	Santa Cruz Biotechnology	Cat#sc-19991
Anti-UCP1 antibody	Abcam, Cambridge	Cat#ab10983
Anti-BCKDK antibody	Abcam	Cat#ab151297
Anti-BCKDHA antibody	Abcam	Cat#ab138460
Anti-BCKDHA phosphor S293 antibody	Abcam	Cat#ab200577
Anti-GAPDH antibody	Cell Signaling Technology	Cat#14C10
Anti-CD16/CD32 antibody	BD Biosciences	Cat#553142
Anti-CD45 antibody	BD Biosciences	Cat#557659
Anti-F4/80 antibody	BD Biosciences	Cat#565411
Anti-CD11b antibody	BD Biosciences	Cat#563402
Anti-Ly6G antibody	BD Biosciences	Cat#560602

**Bacterial and virus strains**		

*Bacteroides dorei*	Deutsche Sammlung von Mikroorganismen	Cat#17855
*Bacteroides vulgatus*	American Type Culture Collection	Cat#8482

**Biological samples**		

Human body fluids (feces, plasma)	This paper	N/A
Mice feces	This paper	N/A
Mice brown adipose tissue	This paper	N/A

**Chemicals, peptides, and recombinant proteins**		

Recombinant murine TNF-α	PeproTech	Cat#315-01A
3,6-dichlorobenzo[b]thiophene-2-carboxylic acid	Cayman Chemical Company	Cat#22948
Noradrenaline	Sigma-Aldrich	Cat#A7257
L-Valine, [1-^14^C]	Moravek, Inc.	Cat#MC-1133

**Critical commercial assays**		

Human Adiponectin ELISA Kit	Otsuka Pharmaceutical Co., Ltd.	N/A
Human Leptin Immunoassay	R&D Systems, Inc.	Cat#DLP00
Limulus Amebocyte Lysate Assay	Lonza Inc.	Cat#K50-643J

**Deposited data**		

Metagenome sequencing data and 16S rRNA sequencing of fecal samples from human subjects and mice	DNA Data Bank of Japan	BioProject database; accession numbers PRJDB7630 and PRJDB8678

**Experimental models: Cell lines**		

HB2 brown preadipocyte	(Irie et al., 1999).	N/A
RAW 264.7	(Raschke et al., 1978)	N/A

**Experimental models: Organisms/strains**		

Mouse C57BL/6J (specific pathogen-free)	CLEA Japan	N/A
Mouse C57BL/6J (germ-free)	CLEA Japan	N/A

**Oligonucleotides**		

Mouse TNF-α	Thermo Fisher Scientific	Forward: AAA ATT CGA GTG ACA AGC CTG TA
		Reverse: CCC TTG AAG AGA ACC TGG GAG TA
Mouse F4/80	Thermo Fisher Scientific	Forward: CTT TGG CTA TGG GCT TCC AGT C
		Reverse: GCA AGG AGG ACA GAG TTT ATC GTG
Mouse GAPDH	Thermo Fisher Scientific	Forward: TGT GTC CGT CGT GGA TCT GA
		Reverse: TTG CTG TTG AAG TCG CAG GAG

**Software and algorithms**		

GraphPad Prism v7.0	GraphPad Software Inc.	https://www.graphpad.com/
JMP v14	SAS Institute	https://www.sas.com/en_us/home.html
R software v3.1.0	R Core Team	https://www.r-project.org/
ImageJ	(Schneider et al., 2012)	https://imagej.net/ImageJ

**Other**		

High-Fat Diet	Oriental Yeast Co., Ltd.	HFD-60

### Resource availability

#### Lead contact

Further information and requests for resources should be directed to and will be fulfilled by the Lead Contact, Tomoya Yamashita (tomoya@med.kobe-u.ac.jp).

#### Materials availability

This study did not generate new unique reagents.

### Experimental model and subject details

#### Clinical study

Fifteen patients with obesity scheduled to undergo LSG were recruited to participate in the study at Chibune General Hospital (Osaka, Japan) between May 2019 and October 2019. Patients who were treated with antibiotics within 4 weeks before the study commenced were excluded from the study. The blood samples from fasting patients were collected in ethylenediaminetetraacetic acid-coated tubes, centrifuged at 4°C for 10 min at 1,500 × *g*, and stored at −80°C until further use. Fecal samples for gut microbial analysis were collected in plastic tubes with guanidine thiocyanate buffer (#FS-0007; TechnoSuruga Laboratory Co., Ltd., Shizuoka, Japan) before and 3 months after LSG, and stored at −80°C until use. Fecal samples from seven obese participants for culturing in the KUHIMM were collected before LSG in an anaerobic culture swab transport system (#212550; Becton, Dickinson and Company, Franklin Lakes, NJ, USA). We also used clinical and metabolome data who were hospitalized at Kobe University Hospital (Kobe, Japan) and had hypertension, diabetes mellitus, or dyslipidemia, as non-obese controls. All participants provided oral and written informed consent, and the study was conducted according to the principles of the Declaration of Helsinki. This study was approved by Kobe University (no. 180355 and B200127) and Chibune General Hospital's Ethics Committee (no. 20190422A), and was registered with the UMIN Clinical Trials Registry (no. UMIN000035635).

#### Animal study

All mice used in the present study was male mice. Specific pathogen-free C57BL/6 male mice were purchased from CLEA Japan (Tokyo, Japan) and housed in a specific pathogen-free animal facility at the Kobe University Institute. The animals were fed a standard chow diet (CE-2; CLEA) or a HFD with 60 kcal % fat (HFD-60; Oriental Yeast Co., Ltd., Tokyo, Japan). Germ-free C57BL/6 male mice were maintained in gnotobiotic isolators (CLEA) and fed autoclaved CE-2 (0–3 weeks of age), CL-2 (CLEA; 4–5 weeks of age), and HFD-60 (≥ 6 weeks of age). For the gut microbiota study, mice in the *Bacteroides* group were gavaged with live *B. dorei* (DSM #17855; Deutsche Sammlung von Mikroorganismen, Braunschweig, Germany) and *B. vulgatus* (ATCC #8482; American Type Culture Collection, Manassas, VA) at a dose of 2.5 × 10^9^ cfu/100 μL of *B. dorei* and *B. vulgatus*, five times per week, while mice in the control group were gavaged with culture medium. For the BCKDK inhibition study, BT2 (3,6-dichlorobenzo[b]thiophene-2-carboxylic acid; #22948, Cayman Chemical Company, Ann Arbor, MI) was dissolved in DMSO and then diluted in buffer (5% DMSO, 10% cremophor EL, and 85% of 0,1 M sodium bicarbonate, pH 9.0) for delivery (Sun et al., 2016). Mice in the BT2 group were dosed with 40 mg/k BT2 six times per week by oral gavage. Mice in the control group were gavaged with 200 μL of vehicle buffer containing the same components without BT2. All mice were provided water *ad libitum* under a strict 12-h light cycle. All experiments were performed according to the Guidelines for Animal Experiments in effect at Kobe University School of Medicine (guideline no. P180402, P181207, and P210201).

### Method details

#### Plasma biochemical parameters

Plasma adiponectin and leptin levels were analyzed using the human adiponectin ELISA kit (Otsuka Pharmaceutical Co., Ltd., Tokyo, Japan) and the human leptin immunoassay (#DLP00; R&D Systems, Inc., MN, USA), respectively, according to manufacturers’ instructions.

Plasma LPS levels were determined using a limulus amebocyte lysate assay (#K50-643J; Lonza Inc., Basel, Switzerland) according to manufacturer’s instructions. The plasma was diluted 10-fold in pyrogen-free water and inactivated for 15 min at 90°C. LPS measurements were performed in pyrogen-free glass tubes, Eppendorf tubes, and plates.

#### Metagenomic analysis of human gut microbiota

DNA extraction was performed by Nihon Gene Research Laboratories Inc. (Miyagi, Japan) according to previous methods (Furet et al., 2009). Libraries from each sample were prepared using the Rubicon ThruPLEX DNA-Seq Kit (#2010-600, MP Biomedicals, CA, USA) with a unique dual index adapter pair for each sample. Samples were sequenced in a 2 × 150-base pair (bp) paired-end format using NovaSeq 6000 at Takara Bio. Inc. (Shiga, Japan).

Raw reads were processed using fastp v0.21.0 (Chen et al., 2018) with default parameters to remove low-quality regions. To remove reads from the human genome, the reads mapped to GRCh38.p7 using bowtie2 v2.2.9 with default parameters. To estimate species abundance, we used mOTU2 version 2.5.1 (Milanese et al., 2019) and MetaPhlAn version 2.2.0 (Truong et al., 2015) with default parameters.

#### Fecal culturing in KUHIMM

We used a small-scale multi-channel fermenter (Bio Jr.8; ABLE, Tokyo, Japan) composed of eight parallel and independent anaerobic culturing vessels called KUHIMM (Takagi et al., 2016; Yoshida et al., 2020b). Each vessel contained 100 mL of Gifu anaerobic medium (Nissui Pharmaceutical Co., Tokyo, Japan) at pH 6.5. The medium was maintained at 37°C with regular stirring at 300 rpm. Continuous in-flow (15 mL/min) of a filtered N_2_:CO_2_ (80:20) gas maintained anaerobiosis.

#### Non-targeted metabolomics using CE-TOFMS

Peripheral venous blood samples (human) or cardiac blood (mice) were collected in tubes containing EDTA-2Na and immediately centrifuged at 1,200 × *g* and 4°C for 10 min to obtain plasma. Interscapular BAT was harvested from mice and frozen in liquid N_2_. These samples were stored at −80°C until CE-TOFMS analysis by Human Metabolome Technologies (Ohashi et al., 2008). CE-TOFMS analysis was carried out using an Agilent capillary electrophoresis system (Agilent Technologies, Santa Clara, CA). The systems were connected by a fused silica capillary (50 μm i.d. × 80 cm total length) with commercial electrophoresis buffer (H3301-1001 and H3302-1021 for cation and anion analysis, respectively; Human Metabolome Technologies) as the electrolyte. The spectrometer was scanned from *m/z* 50 to 1,000. The peaks were extracted using MasterHands automatic integration software version 2.17.1.11 (Keio University, Yamagata, Japan) to obtain peak information including *m/z*, peak area, and migration time. The areas of the annotated peaks were then normalized based on internal standard levels and sample amounts to obtain the relative level of each metabolite. Some metabolites were evaluated by absolute quantification.

#### BCAA and BCKA quantification using LC-MS

The LC-MS system consisted of a Q-Trap 6500 (Sciex, Framingham, MA, USA) equipped with a Shimadzu LC-30AD HPLC system (Shimadzu Corporation, Kyoto, Japan). The stable isotope-labeled internal standards APDSTAG® Amino Acids Internal Standard Mixture Solution (FujiFilm-Wako, Osaka, Japan) and KIV-^13^C_5_ (Cambridge Isotope Laboratories, Inc., Tewksbury, MA, USA) were added to the samples to facilitate quantification. For BCAA analysis, an Intrada Amino Acid column (100 × 3 mm, 3.0 μm; Imtakt, Kyoto, Japan) was used with an acetonitrile/100 mM ammonium formate/formic acid gradient of 85:15:0.1 to 0:100:0 (v/v/v) and a flow rate of 0.6 mL/min. For BCKA analysis, an Intrada Organic Acid column (150 mm × 2 mm, 3.0 μm; Imtakt) was used with an acetonitrile/water/100 mM ammonium formate/formic acid gradient of 10:90:0:0.1 to 10:0:90:0 (v/v/v/v) and a flow rate of 0.2 mL/min. For monitoring and quantifying BCAA and BCKA levels, a multiple reaction monitoring (MRM) method was developed with signature ion pairs Q1 (parent ion)/Q3 (characteristic fragment ion).

#### Culture and preparation of B. dorei and B. vulgatus

*Bacteroides dorei* and *B. vulgatus* were cultured anaerobically in Difco^TM^ reinforced clostridial medium (#218081; BD Bioscience, San Jose, CA) at 37°C. An anaerobic chamber (Coy Laboratory Products, Grass Lake, MI) with 10% CO_2_, 10% H_2_, and 80% N_2_ was used for all anaerobic microbiology steps.

#### Analysis of mouse fecal samples

Fecal samples from mice were collected at 18 weeks of age. DNA extraction, 16S rRNA gene amplification, and sequencing using the MiSeq system (Illumina, San Diego, CA) were performed by TechnoSuruga Laboratory Co., Ltd. as previously described (Takahashi et al., 2014). Briefly, frozen fecal samples were beaten with zirconia beads and DNA was extracted. 16S rRNA genes (the V3–V4 region) were polymerase chain reaction–amplified. After addition of the sequencing adapters, the amplicons were sequenced using an Illumina MiSeq platform (Illumina, San Diego, CA). Analyses of sequence reads were performed manually using the Ribosomal Database Project Multiclassifier tool. Functional analysis of gut microbiota based on the KEGG Orthology database (Kyoto Encyclopedia of Genes and Genomes) (Kanehisa and Goto, 2000) was performed using PICRUSt1.1.1.

#### Mouse BAT RNA sequencing and functional analysis

Total RNA was extracted from the tissue or cell samples using TRIzol reagent (#15596018; Thermo Fisher Scientific, Waltham, MA, US) according to manufacturer’s instructions. The SMART-Seq® v4 Ultra® Low Input RNA Kit for Sequencing (Clontech Laboratories Inc., San Francisco, CA), Nextera XT DNA Library Preparation Kit (Illumina), and IDT for Illumina DNA/RNA UD Indexes (Illumina) were used for next-generation sequencing library construction according to the manufacturer’s protocols. Samples were sequenced in a 2 × 150-bp paired end format using the Illumina NovaSeq 6000 at Takara Bio Inc. The clean data for each sample were aligned to the GENCODE reference gene set (GRCm38.primary_assembly.genome.fa.gz) using DRAGEN Bio-IT Platform (v3.6.3). The read count data were uploaded to iDEP.91 (Ge et al., 2018). After data filtering (0.5 counts per million in at least one sample), we performed principal component analysis. For differential expression analysis, we used DESeq2 to normalize the data to identify differentially expressed genes (DEGs). Genes in the two groups with |log_2_fold change| > 1 and q-value < 0.05 were defined as DEGs. GO enrichment (Ashburner et al., 2000) and KEGG pathway (Kanehisa and Goto, 2000) analyses of DEGs were then performed. Heat maps of the cluster analysis with a dendrogram were plotted using MultiExperiment Viewer (MeV) (Howe et al., 2011).

#### Oral glucose tolerance and insulin tolerance tests

Glucose (1 g/kg body weight) was gavaged and insulin (1 U/kg body weight) was administered intraperitoneally. Blood glucose levels were determined at various time points using a glucometer (Medisafe FIT®; Terumo, Tokyo, Japan) after the initial injection of glucose or insulin.

#### Temperature recording

Mice were transferred to a cold chamber (8°C; #HC-10; SHINFACTORY, Fukuoka, Japan) with individual housing and 5 g bedding for up to 7 h. Food or water was not restricted. Rectal body temperature was obtained using a BAT-10 microprobe thermometer (Physitemp Instruments, Inc., Clifton, NJ) with a rectal probe for mice (#RET-3; Physitemp Instruments). Tissue temperature was also recorded using a BAT-10 Microprobe Thermometer (Physitemp Instruments) with type T thermocouple probes (#IT-18; Physitemp Instruments) in the interscapular BAT. When tissue temperature was stable, mice were intraperitoneally administered noradrenaline (#A7257; Sigma-Aldrich, St. Louis, MO) at a dose of 1 mg/kg to induce thermogenesis.

#### Surgical removal of interscapular BAT

The mice were anesthetized using three types of mixed anesthesia to remove the interscapular BAT through a small longitudinal incision between the shoulder blades. The Sulzer’s vein draining the interscapular BAT was tied off with a suture and cut. The two interscapular BAT lobes were quickly and completely removed. The incision was closed and the mice were allowed to recover. Mice that had been subjected to sham surgery were anesthetized and incised but no manipulation of removal of interscapular BAT was performed.

#### Analysis of short-chain fatty acids in fecal samples

Fecal short-chain fatty acids were measured by TechnoSuruga Laboratory Co., Ltd. as previously described (García-Villalba et al., 2012). Briefly, 0.1 g of feces was resuspended in 0.9 mL of 0.5% phosphoric acid with zirconia beads. Samples were heated at 85°C for 15 min, vortexed at 5 m/s for 45 s using FastPrep 24 (MP Biomedicals, Santa Ana, CA), and centrifuged at 15,000 × *g* for 10 min. Next, 0.4 mL of the supernatant was mixed with 0.4 mL ethyl acetate, shaken for 30 min, and centrifuged at 15,000 × *g* for 10 min. The supernatant (0.2 mL) was then mixed with 1 mM 4-methyl valeric acid as an internal standard. Short-chain fatty acids in feces were measured using gas chromatography with a flame ionization detector (7890B; Agilent Technologies) and the capillary column DB-WAXetr (30 m, 0.25 mm id, 0.25 μm film thickness; Agilent Technologies).

#### Histological analysis

Paraffin-embedded slides were deparaffinized in xylene, rehydrated in ethanol, and treated with microwave-based antigen retrieval, followed by 1% hydrogen peroxide. For picrosirius red staining, we used the Picrosirius Red Stain Kit (#24901; Polysciences Inc., Warrington, PA). For MAC3 staining, sections were incubated overnight at 4°C with rat-anti-mouse LAMP-2 (M3/84) antibody (#sc-19991; 1:100; Santa Cruz Biotechnology, Santa Cruz, CA), and rabbit-anti-mouse UCP1 antibody (#ab10983; 1:500; Abcam, Cambridge, UK), followed by detection with secondary antibodies (#424141; NICHIREI Biosciences Inc., Tokyo, Japan) and DAB-peroxidase (#425011, NICHIREI Biosciences Inc.). Stained sections were digitally captured using an all-in-one fluorescence microscope (BZ-8000; Keyence, Tokyo, Japan).

#### Western blotting

The cells were rinsed twice with PBS and harvested in ice-cold RIPA Buffer (#16488-34; Nacalai Tesque, Kyoto, Japan) with phosphatase inhibitor cocktails (#07574-61, Nacalai Tesque). Proteins isolated from total lysates were separated via 10% SDS-PAGE and transferred onto polyvinylidene difluoride membranes (#IB401002; Thermo Fisher Scientific) using an iBlot Gel Transfer Device (Thermo Fisher Scientific). The membranes were blocked in 5% milk (#31149-75; Nacalai Tesque) and incubated with primary antibodies overnight at 4°C, followed by incubation with horseradish peroxidase-conjugated secondary antibodies (#6721; 1:5,000; Abcam) for 1 h at room temperature. After incubation with Immobilon Western HRP Substrate (#WBKLS0500; Merck Millipore, Billerica, MA), the signals were detected using the V3 Western Workflow system (Bio-Rad Laboratories, Hercules, CA). The following primary antibodies were used in this study: Anti-BCKDK (#ab151297; 1:3,000; Abcam), anti-BCKDHA (#ab138460; 1:5,000; Abcam), anti-BCKDHA (phosphor S293; #ab200577; 1:5,000; Abcam), and anti-GAPDH (#14C10; 1:6,000; Cell Signaling Technology, Danvers, MA). The relative protein levels were semi-quantified using the ImageJ® software (https://imagej.net/ImageJ).

#### *In vitro* experiments with the HB2 cell line

The HB2 brown preadipocyte (provided by Dr. Okamatsu-Ogura Yuko and Dr. Masayuki Saito, Hokkaido University, Sapporo, Japan) (Irie et al., 1999) and RAW 264.7 macrophage cell lines were maintained in DMEM (#D6046; Sigma-Aldrich) supplemented with 10% fetal bovine serum (FBS) at 37°C in a humidified 5% CO_2_ atmosphere. For differentiation of HB2 cells, confluent cells were cultured in induction medium composed of DMEM supplemented with 10% FBS, 0.5 mM 3-isobutyl-1-methylxanthine (#I5879; MilliporeSigma, Burlington, MA), and 1 μM dexamethasone (#D2915; MilliporeSigma) for the first 2 days. Then, cells were cultured for an additional 2 days in differentiation medium composed of DMEM supplemented with 10% FBS, 10 μg/mL insulin (#0105; Cell Science & Technology Institute, Inc., Sendai, Japan), and 50 nM of 3,3,5-triiodo-L-thyronine (#T5516; MilliporeSigma). In the co-culture system, serum-starved differentiated HB2 cells were cultured in a 6-cm dish, and RAW264.7 (10^6^ cells/mL) were plated onto HB2 cells. Recombinant murine TNF-α (#315-01A; PeproTech, Rocky Hill, NJ) was used for stimulation. The cells were cultured for 24 h in contact with each other and then harvested.

Valine oxidation assays were performed as previously described (Yoneshiro et al., 2019). Briefly, differentiated HB2 cells in a 6-well plate were washed with PBS and incubated in 1 mL KRP-HEPES buffer containing 0.16 μCi/mL [1-^14^C] valine at 37°C for 2 h. Subsequently, 300 μL of 30% hydrogen peroxide was added in each well, and [^14^C] CO_2_ was trapped in the smears supplemented with 300 μL of 1 M benzethonium hydroxide solution for 20 min. Valine oxidation was quantified by counting radioactivity of trapped [^14^C] CO_2_ using a scintillation counter.

#### BAT BCAA oxidation assays

BAT BCAA oxidation assays were performed as described previously (Yoneshiro et al., 2019). Briefly, 50 mg of the isolated BAT was minced carefully using scissors for 3 min, placed in a polypropylene round-bottom tube, and incubated in 1 mL KRP–HEPES buffer containing 0.16 μCi/mL [1-^14^C] valine at 37°C for 1 h. Subsequently, 300 μL of 30% hydrogen peroxide was added to the tube and [^14^C]CO_2_ was trapped in the smears placed in the center well supplemented with 300 μL of 1 M benzethonium hydroxide for 20 min. Valine oxidation was quantified by calculating the radioactivity of the trapped [^14^C] CO_2_ using a scintillation counter.

#### Real-time PCR

Total RNA was extracted from the tissue or cell samples using TRIzol reagent according to manufacturer’s instructions and then reverse transcribed into cDNA using the PrimeScript Reverse Transcription Reagent Kit (#RR037A; Takara Bio). Real-time PCR was performed using SYBR Premix Ex Taq (#RR820; Takara Bio) on a LightCycler® 96 System (#05815916001; Roche, Mannheim, Germany) according to manufacturers’ instructions. Expression data were normalized to GAPDH as a housekeeping gene and analyzed according to the ΔΔCT method.

#### Flow cytometry

Interscapular BAT was dissected from the mice gavaged with *Bacteroides* or vehicle for 12 weeks and processed for cell isolation as described previously (Hagberg et al., 2018). Briefly, 100 mg of BAT was minced carefully using scissors for 3 min. The minced tissue was digested in a 37°C thermal shaker (#0003637; TAITEC, Saitama, Japan) for 30 min with Collagenase Type 1 (#LS004196; Worthington Biochemical Corp., Lakewood, NJ) prepared in 10 mL PBS. The samples were then centrifuged for 2 min at 20 × *g*, and the pellets (stromal vascular fraction) were washed 2 times with 10 mL PBS containing 2% bovine serum albumin and incubated with an anti-CD16/CD32 antibody (#553142; BD Biosciences) to block Fc receptors. This was followed by staining with the following antibodies: anti-CD45 (#557659; BD Biosciences), anti-F4/80 (#565411; BD Biosciences), anti-CD11b (#563402; BD Biosciences), and anti-Ly6G (#560602; BD Biosciences). Samples incubated with the isotype-matched antibodies were used as controls. Flow cytometric analysis was performed on an Attune acoustic focusing cytometer (Life Technologies, Grand Island, NY) and by using FlowJo software (Tree Star, Inc., Ashland, OR).

### Quantification and statistical analysis

Statistical analysis was performed using R software (version 3.1.0; https://www.r-project.org/), JMP (version 14; SAS Institute, Cary, NC), and GraphPad Prism (version 7.0; GraphPad Software Inc., La Jolla, CA). The Shapiro-Wilk test was used to determine whether the data were normally distributed. Results are expressed as mean ± standard error of the mean or standard deviation for normally distributed data, and median ± interquartile range (25^th^–75^th^ percentile) for non-normally distributed data. The significance of differences between two groups was evaluated using the two-tailed Student’s *t*-test for normally distributed data or the Mann-Whitney U-test for non-normally distributed data. The χ^2^ test or Fisher’s exact test was used to compare categorical variables. For all tests, *p* values <0.05 were considered statistically significant. To identify the strength and direction of a link between two parameters, Pearson’s correlation coefficient for normally distributed data, or Spearman’s rank correlation coefficient for non-normally distributed data were calculated. For comparisons of the means of more than two groups, one-way analysis of variance (ANOVA) followed by Tukey’s post-hoc test for normally distributed data or Kruskal–Wallis test followed by Dunn’s post-hoc test for non-normally distributed data was used. Two-way ANOVA followed by post-hoc unpaired *t*-tests with Bonferroni’s correction was used for comparisons of repeated measurements. The *q*-values were calculated using the Benjamini–Hochberg method to adjust the *p* values for multiple comparisons. The Shannon–Wiener index was calculated using the vegan package for R software (version 3.1.0; https://www.r-project.org/).

### Additional resources

Human study was registered with the UMIN Clinical Trials Registry (no. UMIN000035635; https://upload.umin.ac.jp/cgi-open-bin/ctr_e/ctr_view.cgi?recptno=R000040594).

## Data Availability

• All data supporting the findings of our study are available from the Lead Contact, Tomoya Yamashita (tomoya@med.kobe-u.ac.jp). • The sequence data have been deposited in the DDBJ BioProject database (Database: PRJDB7630 and PRJDB8678) (http://trace.ddbj.nig.ac.jp/bioproject/index_e.html).

## Supplemental information

Document S1. Figures S1–S7

Table S1. Changes in the clinical parameters of 15 patients who underwent laparoscopic sleeve gastrectomy, related to Figure 1

Table S2. Alterations in the levels of plasma metabolites after laparoscopic sleeve gastrectomy, related to Figure 1

Table S3. Alterations in the levels of BAT metabolites in DIO mice following BT2 treatment, related to Figure 2

Table S4. Alterations in the levels of plasma metabolites in DIO mice following Bacteroides treatment, related to Figure 4

Table S5. Alterations in the levels of BAT metabolites in DIO mice following Bacteroides treatment, related to Figure 4
