# Supplementary material for: Bacteroides spp. promotes branched-chain amino acid catabolism in brown fat and inhibits obesity
Source: iScience. 2021 Oct 24;24(11):103342. doi: 10.1016/j.isci.2021.103342 (PMC8586802; doi:10.1016/j.isci.2021.103342)
Supplement: Document S1. Figures S1–S7 [file mmc1.pdf]

iScience, Volume *24*

Supplemental information

Bacteroides spp. promotes branched-chain amino acid catabolism in brown fat and inhibits obesity

Naofumi Yoshida, Tomoya Yamashita, Tatsunori Osone, Tetsuya Hosooka, Masakazu Shinohara, Seiichi Kitahama, Kengo Sasaki, Daisuke Sasaki, Takeshi Yoneshiro, Tomohiro Suzuki, Takuo Emoto, Yoshihiro Saito, Genki Ozawa, Yushi Hirota, Yasuyuki Kitaura, Yoshiharu Shimomura, Yuko Okamatsu-Ogura, Masayuki Saito, Akihiko Kondo, Shingo Kajimura, Takeshi Inagaki, Wataru Ogawa, Takuji Yamada, and Ken-ichi Hirata

Supplemental Figure Legends

Figure S1. Comparisons of plasma BCAA and BCKA levels between non-obese and obese patients (related to Figure 1).

Data from 11 non-obese controls (UMIN000022414) compared with those from 15 patients with obesity (UMIN000035635). (A) Patient characteristics. (B) Comparisons of plasma Val and KIV concentrations in the two groups. (C) Correlation between BMI and the plasma Val and KIV levels.

Data are shown as mean ± SD (A) or mean ± SEM (B); two-tailed unpaired Student's *t*-test (B); Pearson's correlation coefficient (C). ***$p < 0.001$. BMI, body mass index; KIV, ketoisovaleric acid; Val, valine.

Figure S2. Effects of the BCAA catabolism enhancer on the gastrocnemius muscle and WAT in DIO mice (related to Figure 2).

(A) Weights of the gastrocnemius muscle, inguinal WAT, and epididymal WAT. (B-D) Immunoblotting and quantification of the gastrocnemius muscle (B), inguinal WAT (C), and epididymal WAT (D).

BCKDHA; branched-chain ketoacid dehydrogenase subunit E1α; BT2, 3,6-dichlorobenzo[b]thiophene-2-carboxylic acid; p-BCKDHA, phospho-BCKDHA; WAT, white adipose tissue.

Figure S3. Effects of *Bacteroides* treatment on DIO mice (related to Figure 4).

(A) Experimental design. (B) Plasma lipid profiles. n = 5 per group. (C) Fecal SCFA levels were measured using gas chromatography. n = 7 Ctrl; n = 6 Bac.

Data are shown as mean ± SEM (B) or median ± IQR (C). Two-tailed unpaired Student's t-test (B), Mann–Whitney U-test (C).

HDL, high-density lipoprotein cholesterol; HFD, high-fat diet; LDL, low-density lipoprotein cholesterol; NS, not significant; SCFA, short-chain fatty acids; T-Cho, total cholesterol; TG, triglyceride.

Figure S4. Effect of *Bacteroides* treatment on the white adipose tissue (WAT) (related to Figure 4).

Two *Bacteroides* strains (Bac) or vehicle (Ctrl) were gavaged to C57BL/6 mice fed a high-fat diet five times per week for 12 weeks. C57BL/6 mice were fed a standard chow diet (NC). (A) Adipose tissue weight. n = 4 NC; n = 18 Ctrl; n = 19 Bac. B, C,

Representative HE, MAC3, and Sirius Red staining of iWAT (B) and eWAT (C). Scale bar = 100 μm.

Data are shown as mean ± SEM or median ± IQR; $*p < 0.05$, $**p < 0.01$, $***p < 0.001$. One-way ANOVA followed by Tukey's post-hoc test (A); and two-tailed unpaired Student's *t*-test or two-tailed Mann–Whitney U-test, as appropriate (B, C). iWAT, inguinal white adipose tissue; eWAT, epididymal white adipose tissue; HE, hematoxylin & eosin.

Figure S5. *Bacteroides* treatment alters the gut microbiota in mice (related to Figure 4).

Feces were collected from mice fed a normal chow (NC) or high-fat diet and treated with *Bacteroides* (Bac) or vehicle (Ctrl) for 12 weeks. The V3–V4 region of the bacterial 16S rRNA was sequenced. $n = 4$ NC; $n = 5$ Ctrl; $n = 5$ Bac. (A) Relative abundance of the gut microbiota at the phylum level. (B) Relative abundance of the 20 most abundant microbial genera. (C) Principal component analysis score plots at the genus level. (D) *Firmicutes* to *Bacteroidetes* ratio. (E) Number of bacteria at the genus level. (F) The Shannon-Wiener index at the genus level. (G) Relative abundance of *B. dorei* and *B. vulgatus* (percentage of the total gut microbiota) was assessed via real-time

PCR. (H) The prediction of bacterial BCAA degradation pathway expression based on the 16S rRNA sequences using PICRUSt.

Data are shown as mean ± SEM (E, F, H) or IQR (D, G). $**p < 0.01$, $***p < 0.001$. One-way ANOVA followed by Tukey's post-hoc test (E, F) or Kruskal-Wallis test followed by Dunn's multiple comparison post-hoc test (D, G), and two-tailed unpaired Student's *t*-test (H).

Figure S6. Fluorescence-activated cell sorting (FACS) gating strategy (related to Figure 6).

(A) Representative results of CD45 expression. (B) Representative results of F4/80 expression.

Figure S7. Overview of the metagenomic analysis pipeline and its results (related to Figure 7).

(A) Sample collection and general analysis workflow. (B) Detailed workflow of our metagenome pipeline, including functional annotation and taxonomic annotation. (C–E) Metagenome-based gut microbial composition. Sequence data were annotated with MetaPhlAn2. (C) Cladogram of the gut microbiota. Each dot represents a taxonomic

hierarchy. The green and red dots indicate significant enrichment in the indicated groups [LEfSe: $p < 0.05$, q < 0.1, linear discriminant analysis (LDA) > 2]. Yellow dots represent non-significant differences. (D) Relative abundance of the gut microbiota according to LDA score. (E) Relative abundance of *Bacteroides* spp., *B. dorei*, and *B. vulgatus* before and after LSG. Two-tailed paired *t*-test (E).

A

Variable	Non-obese (n = 11)	Obese (n = 15)
Body weight (kg)<br>BMI (kg/m²)	64.4 ± 13.3<br>24.4 ± 3.1	104. 8 ± 24.0***<br>40.8 ± 6.6***
Comorbidities, n (%)<br>Diabetes mellitus<br>Hypertension<br>Dyslipidemia	 5 (45)<br>9 (82)<br>6 (55)	 10 (67)<br>11 (73)<br>13 (87)

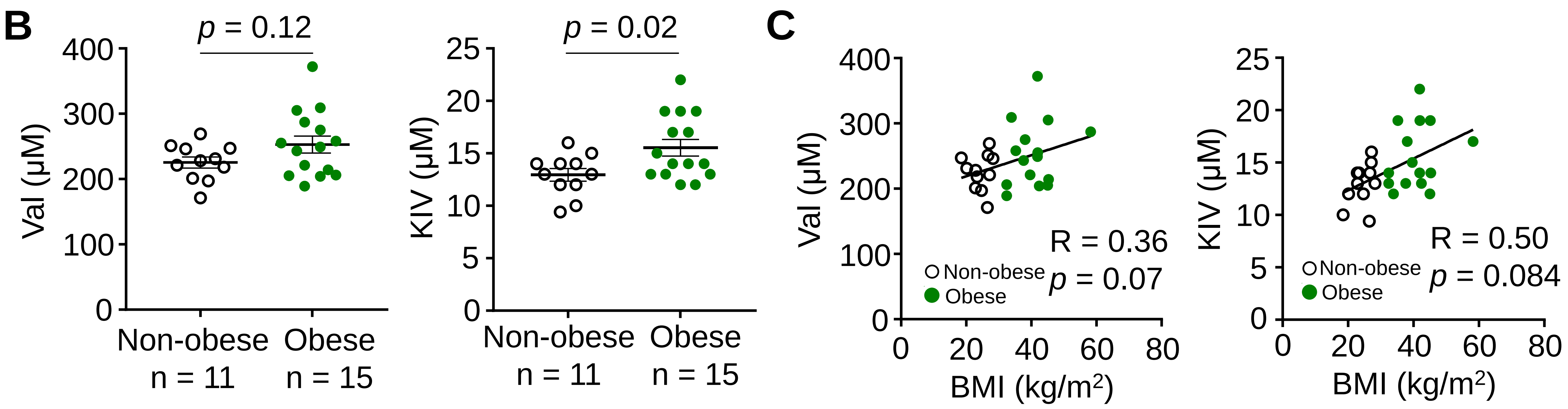

Supplemental Figure 1

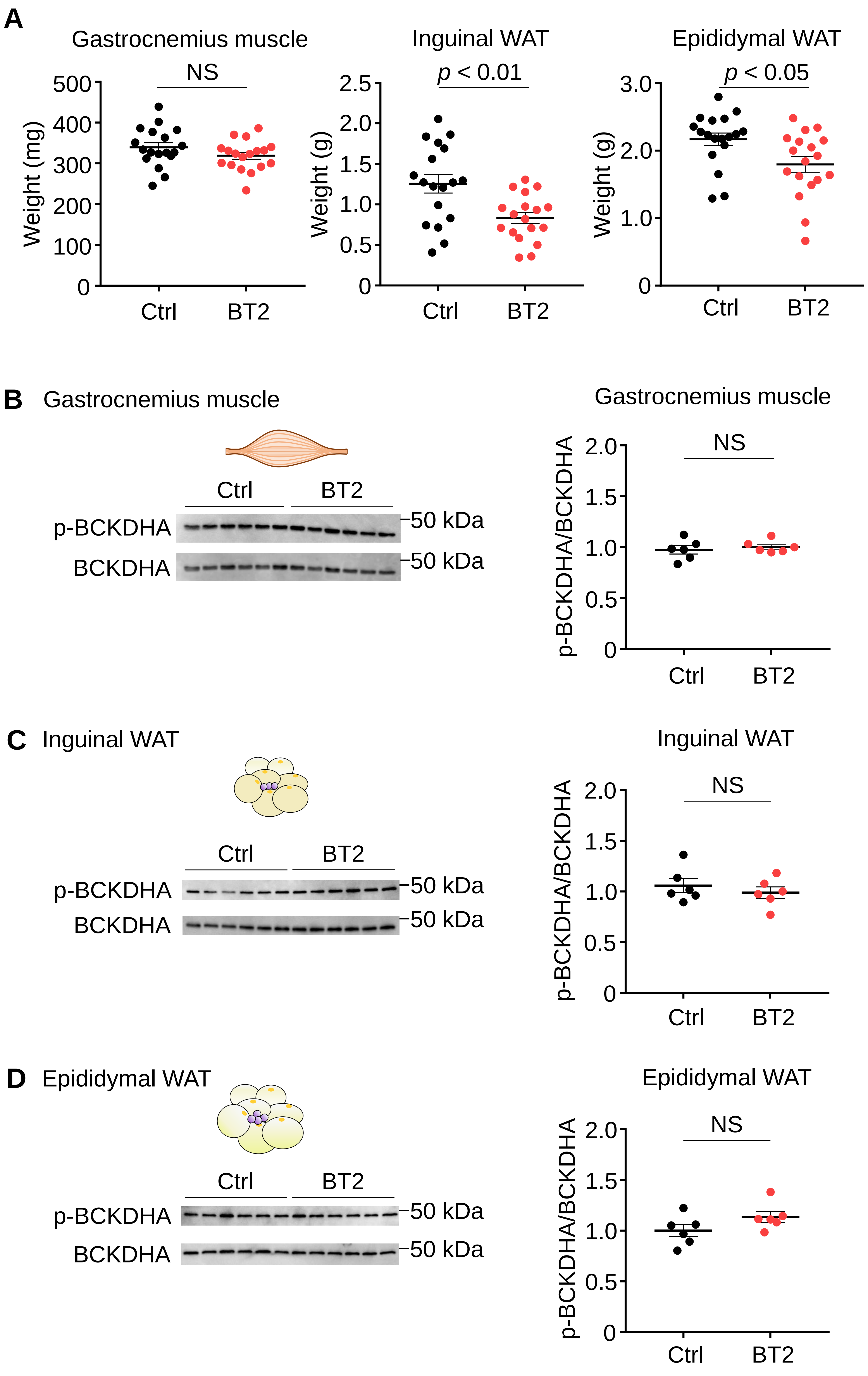

Supplemental Figure 2

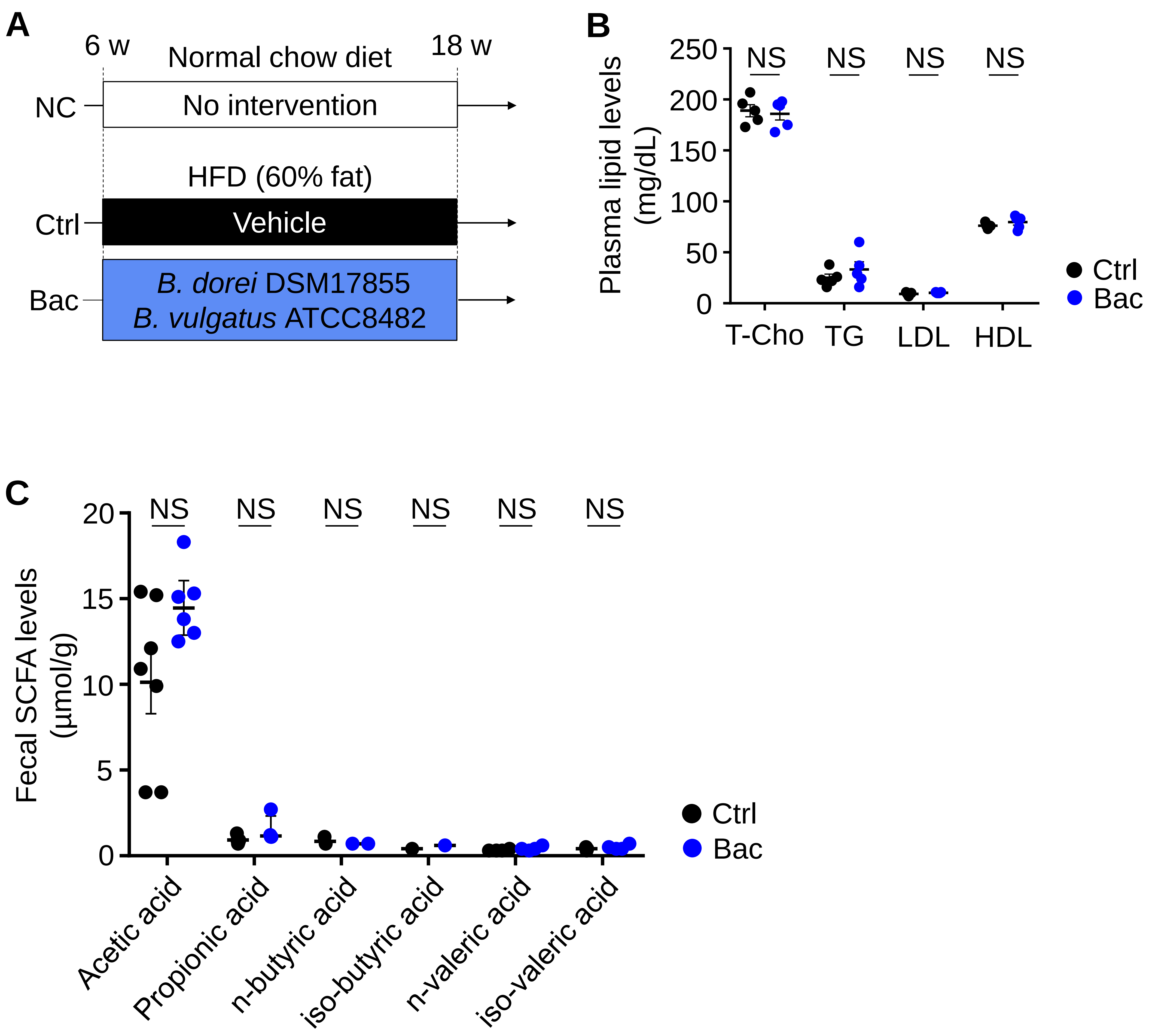

Supplemental Figure 3

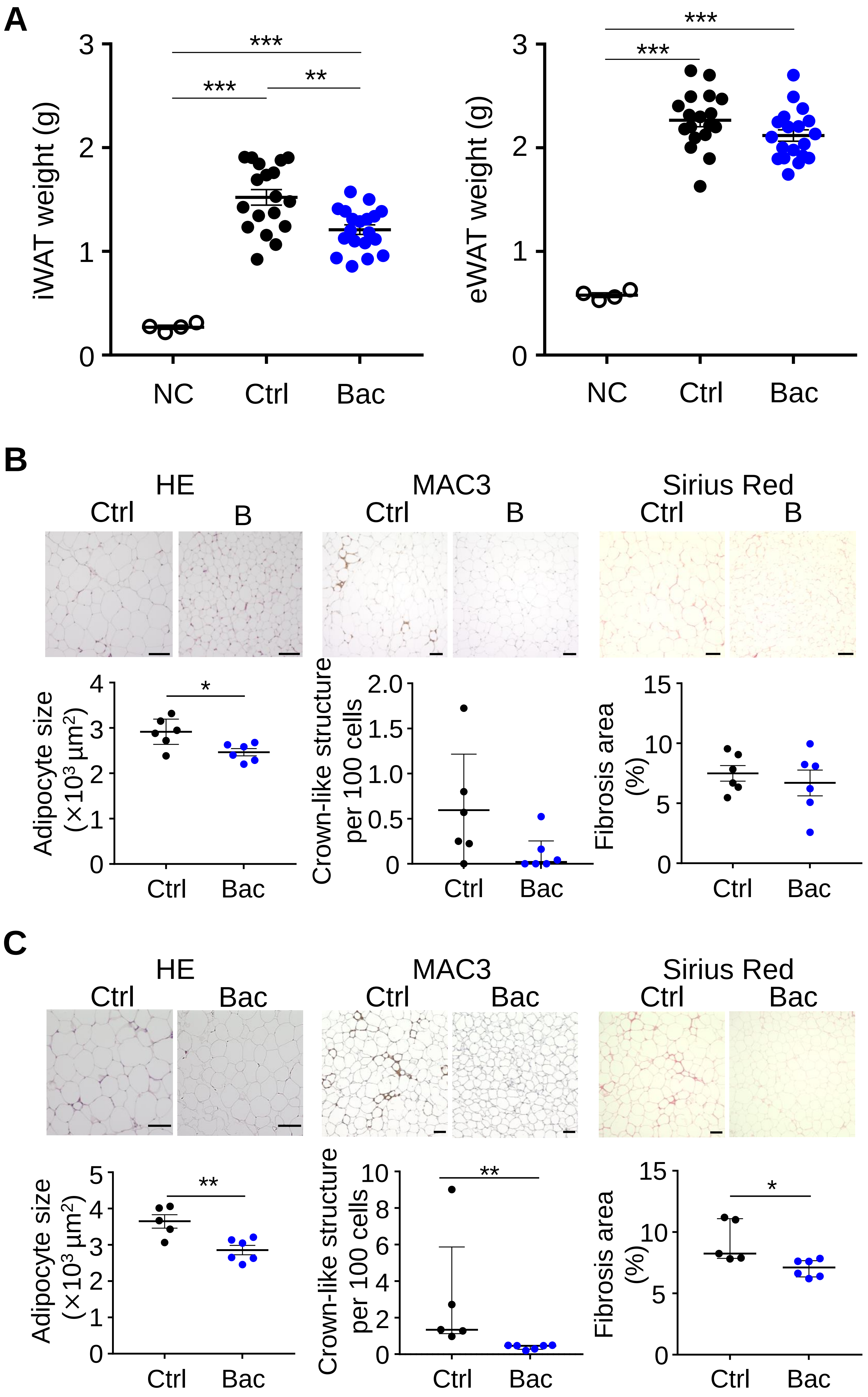

Supplemental Figure 4

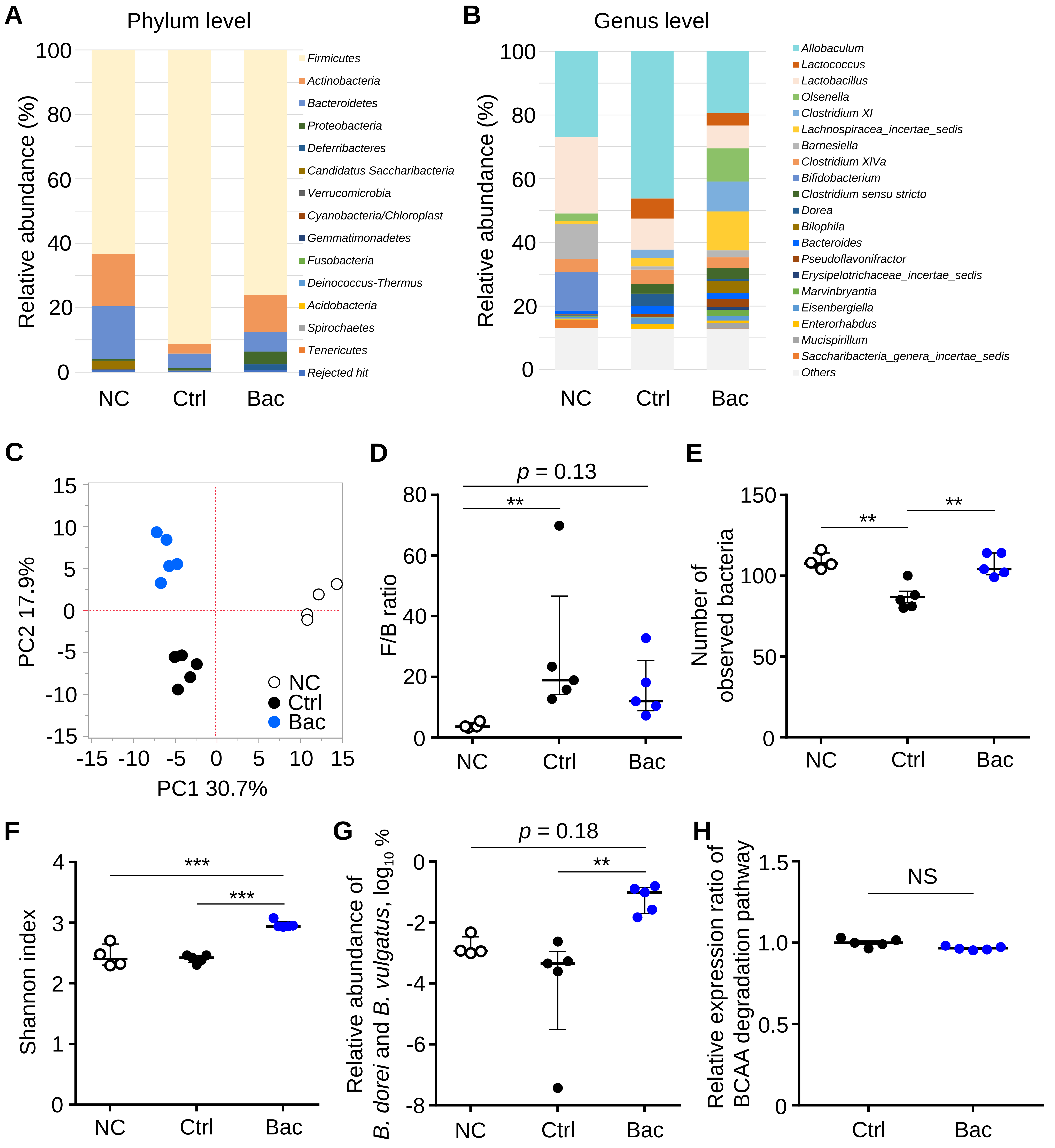

Supplemental Figure 5

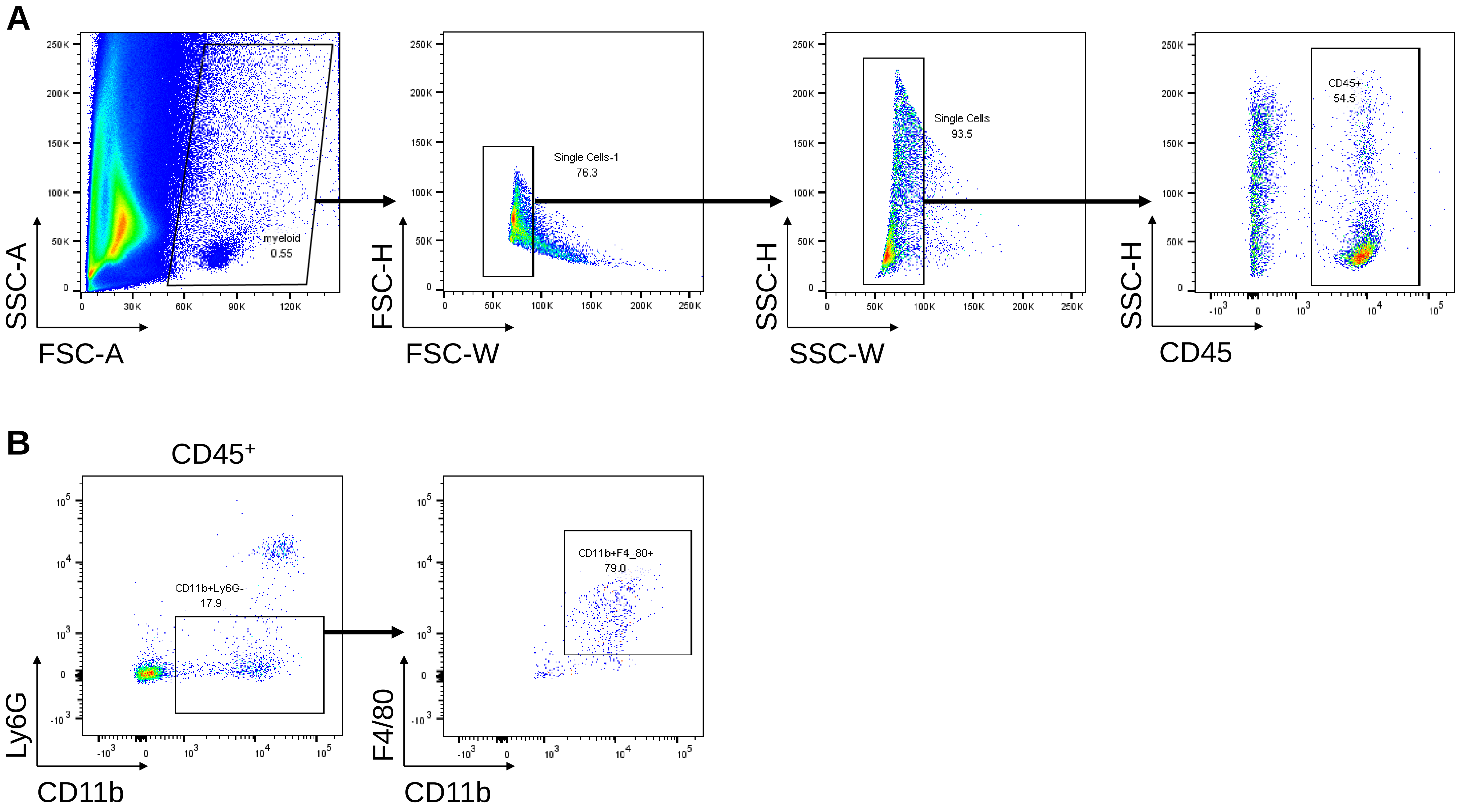

Supplemental Figure 6

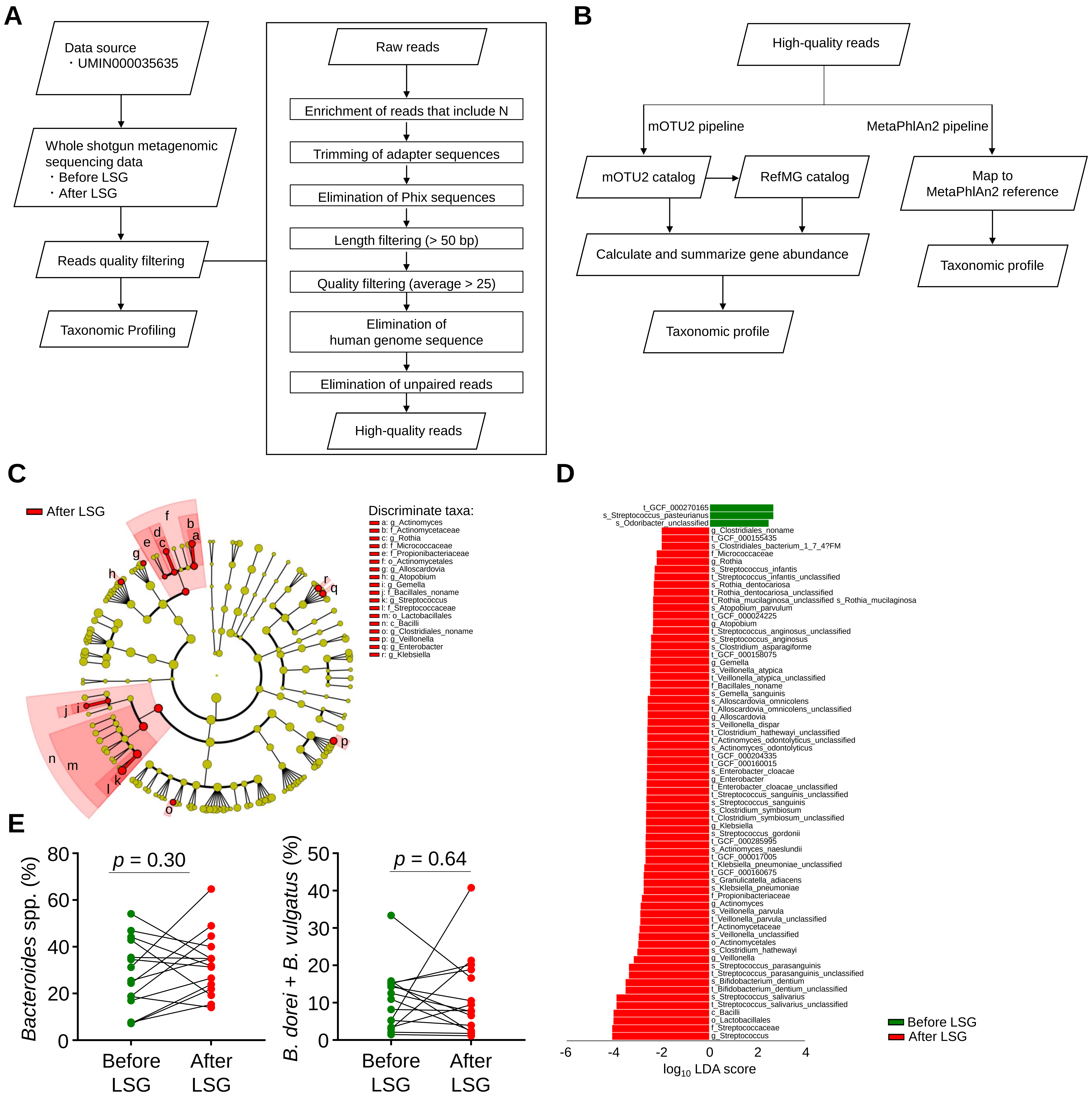

Supplemental Figure 7

Supplemental Table Titles and Legends

Table S1 | Changes in the clinical parameters of 15 patients who underwent laparoscopic sleeve gastrectomy (related to Figure 1).

Table S2. Alterations in the levels of plasma metabolites after laparoscopic sleeve gastrectomy (related to Figure 1).

Table S3. Alterations in the levels of BAT metabolites in DIO mice following BT2 treatment (related to Figure 2).

Table S4. Alterations in the levels of plasma metabolites in DIO mice following *Bacteroides* treatment (related to Figure 4).

Table S5. Alterations in the levels of BAT metabolites in DIO mice following *Bacteroides* treatment (related to Figure 4).
